# *NCYM*, a *Cis*-Antisense Gene of *MYCN*, Encodes a *De Novo* Evolved Protein That Inhibits GSK3β Resulting in the Stabilization of MYCN in Human Neuroblastomas

**DOI:** 10.1371/journal.pgen.1003996

**Published:** 2014-01-02

**Authors:** Yusuke Suenaga, S. M. Rafiqul Islam, Jennifer Alagu, Yoshiki Kaneko, Mamoru Kato, Yukichi Tanaka, Hidetada Kawana, Shamim Hossain, Daisuke Matsumoto, Mami Yamamoto, Wataru Shoji, Makiko Itami, Tatsuhiro Shibata, Yohko Nakamura, Miki Ohira, Seiki Haraguchi, Atsushi Takatori, Akira Nakagawara

**Affiliations:** 1 Division of Biochemistry and Innovative Cancer Therapeutics and Children's Cancer Research Center, Chiba Cancer Center Research Institute, 666-2 Nitona, Chuo-ku, Chiba, Japan; 2 Division of Cancer Genomics, National Cancer Center Research Institute, 5-1-1 Tsukiji, Chuo-ku, Tokyo, Japan; 3 Department of Diagnostic Pathology, Research Institute, Kanagawa Children's Medical Center, 2-138-4 Mutsukawa, Minami-ku, Yokohama, Japan; 4 Division of Surgical Pathology, Chiba Cancer Center, 666-2 Nitona, Chuo-ku, Chiba, Japan; 5 Department of Pediatric Surgery, Graduate School of Medicine, Tohoku University, Sendai, Japan; 6 Laboratory of Cancer Genomics, Chiba Cancer Center Research Institute, 666-2 Nitona, Chuo-ku, Chiba, Japan; Universität Würzburg, Germany

## Abstract

The rearrangement of pre-existing genes has long been thought of as the major mode of new gene generation. Recently, *de novo* gene birth from non-genic DNA was found to be an alternative mechanism to generate novel protein-coding genes. However, its functional role in human disease remains largely unknown. Here we show that *NCYM*, a *cis*-antisense gene of the *MYCN* oncogene, initially thought to be a large non-coding RNA, encodes a *de novo* evolved protein regulating the pathogenesis of human cancers, particularly neuroblastoma. The *NCYM* gene is evolutionally conserved only in the taxonomic group containing humans and chimpanzees. In primary human neuroblastomas, *NCYM* is 100% co-amplified and co-expressed with *MYCN*, and *NCYM* mRNA expression is associated with poor clinical outcome. MYCN directly transactivates both *NCYM* and *MYCN* mRNA, whereas NCYM stabilizes MYCN protein by inhibiting the activity of GSK3β, a kinase that promotes MYCN degradation. In contrast to *MYCN* transgenic mice, neuroblastomas in *MYCN/NCYM* double transgenic mice were frequently accompanied by distant metastases, behavior reminiscent of human neuroblastomas with *MYCN* amplification. The NCYM protein also interacts with GSK3β, thereby stabilizing the MYCN protein in the tumors of the *MYCN*/*NCYM* double transgenic mice. Thus, these results suggest that GSK3β inhibition by NCYM stabilizes the MYCN protein both *in vitro* and *in vivo*. Furthermore, the survival of *MYCN* transgenic mice bearing neuroblastoma was improved by treatment with NVP-BEZ235, a dual PI3K/mTOR inhibitor shown to destabilize MYCN via GSK3β activation. In contrast, tumors caused in *MYCN/NCYM* double transgenic mice showed chemo-resistance to the drug. Collectively, our results show that NCYM is the first *de novo* evolved protein known to act as an oncopromoting factor in human cancer, and suggest that *de novo* evolved proteins may functionally characterize human disease.

## Author Summary

The *MYCN* oncogene has a central role in the genesis and progression of neuroblastomas, and its amplification is associated with an unfavorable prognosis. We have found that *NCYM*, a *MYCN cis*-antisense RNA, is translated in humans to a *de novo* evolved protein. NCYM inhibits GSK3β to stabilize MYCN, whereas MYCN induces *NCYM* transcription. The positive feedback regulation formed in the *MYCN/NCYM*-amplified tumors promotes the aggressive nature of human neuroblastoma. *MYCN* transgenic mice, which express human *MYCN* in sympathoadrenal tissues, spontaneously develop neuroblastomas. However, unlike human neuroblastoma, distant metastasis is infrequent. We established *MYCN*/*NCYM* double transgenic mice as a new animal model for studying neuroblastoma pathogenesis. We found that NCYM expression promoted both the metastasis and chemo-resistance of the neuroblastomas formed in the double transgenic mice. These results demonstrate that NCYM may be a potential target for developing novel therapeutic tools against high-risk neuroblastomas in humans, and that the *MYCN*/*NCYM* double transgenic mouse may be a suitable model for the screening of these new drugs.

## Introduction

Gene evolution has long been thought to arise from pre-existing genes through duplication or rearrangement followed by rapid divergence [1]–[5]. *De novo* gene birth from non-coding genomic regions has been generally believed to be exceptionally rare [1]. However, recent studies using genome-wide analyses have suggested the presence of a large number of *de novo* evolved genes in some species [3], [5]–[11], including primates [12]–[17]. Studies in yeast revealed that the proteins produced from *de novo* genes were not insignificant polypeptides but functional proteins [6], [7] and that *de novo* gene birth could be a major mechanism of new gene generation [6]. In multicellular organisms, however, the functions of *de novo* evolved proteins have been poorly characterized [3], [15], and thus their pathophysiological significance has remained elusive. Therefore, it is still unclear whether *de novo* gene birth is a general mechanism throughout evolution for the creation of functional protein-coding genes.

**Figure 1:**
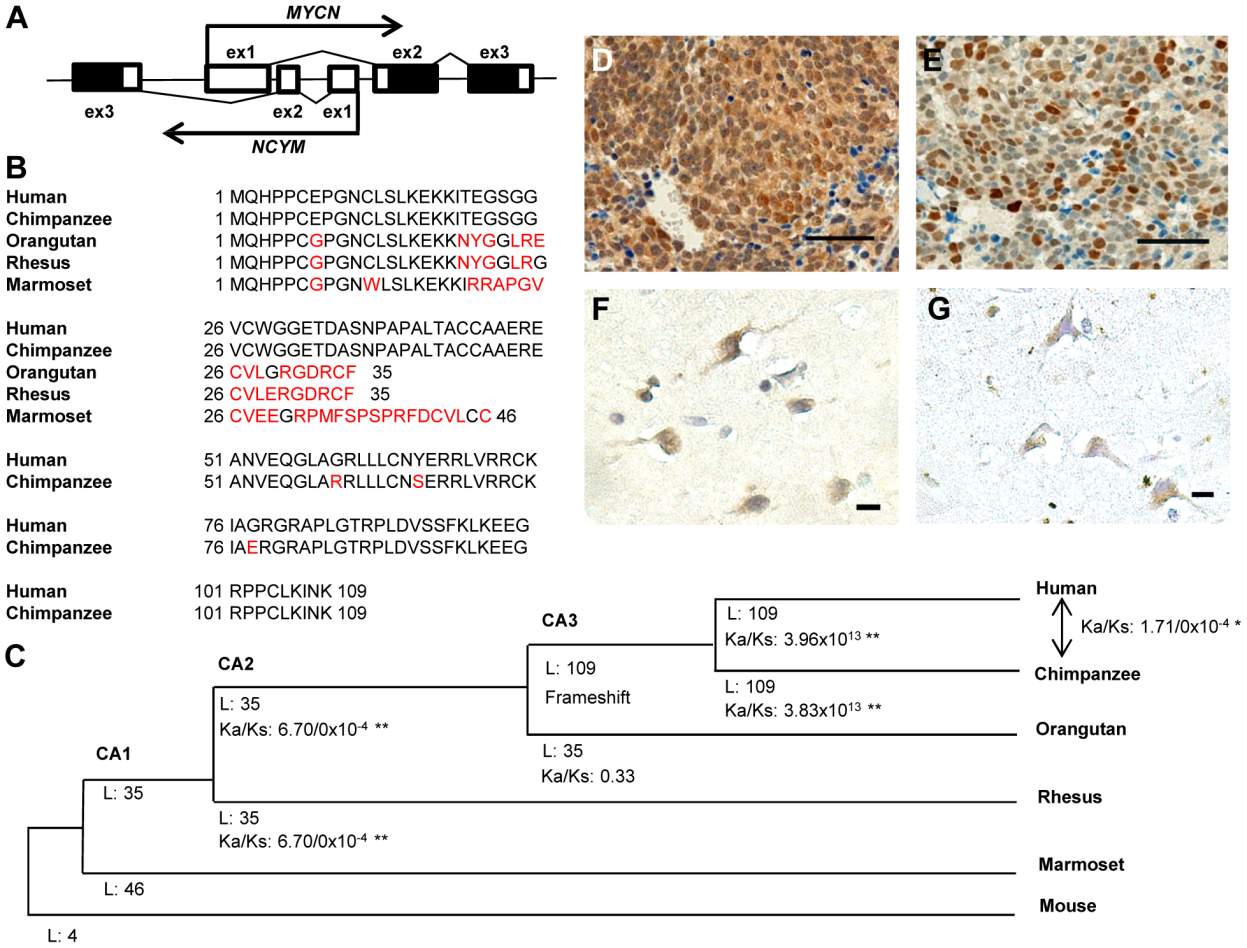
*NCYM* encodes a *de novo* evolved protein in humans. (A) Gene structure of the human *MYCN*/*NCYM* locus. (B) Alignment of the possible amino acid sequences of NCYM in the human and primate genomes, where the ORF of the primate genes begins at the same position as the human start codon. Red text indicates amino acid differences compared with human NCYM. (C) Change in protein features along the lineage shown. CA indicates common ancestor. L indicates the sequence length of amino acids before the first terminal codon. Asterisk indicates statistical significance (**P<0.001, *P<0.05). *K*_a_ and *K*_s_ indicate the rate of non-synonymous changes and synonymous changes, respectively. (D–G) The protein expression of NCYM and MYCN in human primary neuroblastomas (D, E) and normal human cerebrum (F, G). Scale bars, 100 µm (D, E) and 50 µm (F, G). Sections of neuroblastomas with *MYCN* amplification and those of normal human cerebrum were stained with anti-NCYM (D, F) or anti-MYCN (E, G) antibodies.

Neuroblastoma is one of the most common solid tumors in children. It originates from the neuronal precursor cells of the sympathoadrenal lineage of the neural crest [18]. Its clinical behavior is enigmatic; the tumors in patients of less than one year of age often regress spontaneously, whereas the tumors detected in patients over one year of age are usually aggressive and eventually cause the patient's death despite intensive multimodality therapies [18]. The *MYCN* oncogene is frequently amplified in those tumors that occur in patients who are over one year of age at diagnosis [19], [20]. Transgenic mice expressing human *MYCN* in sympathoadrenal tissues spontaneously develop neuroblastomas [21], suggesting that *MYCN* alone can initiate tumorigenesis and promote tumor growth. However, unlike human neuroblastoma, its distant metastasis is infrequent. Furthermore, in human neuroblastomas without *MYCN* amplification, *MYCN* mRNA expression levels do not correlate with the prognosis of the patients [22], [23], suggesting that additional events might contribute to the acquisition of increased aggressiveness. We focused on *NCYM* as a candidate gene that promotes the aggressiveness of *MYCN*-amplified neuroblastomas. *NCYM* is a *cis*-antisense gene of *MYCN* [24], [25] and is co-amplified with *MYCN* in human neuroblastoma cells. *NCYM* is transcribed in the opposite direction to *MYCN*, starting from intron 1 of the *MYCN* gene (Figure 1A), and it has remained unclear for a long time whether the gene encodes a functional protein [24], [26]. In this study, we have found that NCYM is indeed a functional protein that regulates MYCN function in human, but not mouse, neuroblastoma.

## Results

### *NCYM* is a *de novo* evolved gene

We first analyzed the genomic sequence of *NCYM* in various species and found that in humans and chimpanzees the potential NCYM protein is composed of 109 amino acids (Figure 1B, Figure S1). We next searched for paralogs and orthologs of the human NCYM protein among other animals using the Basic Local Alignment Search Tool (BLAST) databases with an E-value threshold of 10^−3^. We did not find any paralogs, but identified orthologs for a probable NCYM protein in olive baboons, chimpanzees and pigmy chimpanzees. From here on, we focused on the *NCYM* gene of the hominidae to investigate the function of the protein product. The evolutionary rates between the indicated species suggest that the coding sequence of *NCYM* gene was exposed to positive selection in humans and chimpanzees (Figure 1C), and the amino acid frequencies in these species were significantly different from the uniform usage of amino acids (*P*<0.001; Figure S2). We next raised an antibody against the putative human NCYM protein, and identified a 12 to 15 kDa protein in human neuroblastoma cells which mainly localized to nuclei in *MYCN*-amplified neuroblastoma cells (Figure S3, Figure S4). The NCYM protein was expressed in a variety of normal human tissues, including the neuronal cells of the cerebrum and cerebellum, spermatocytes of the testis, pancreatic cells and also the heart (Figure S5). NCYM was also localized in both the nucleus and cytoplasm in these cells (Figure S5A–D). NCYM was expressed in both primary and metastatic human neuroblastomas (Figure 1D, Figure S5E and F), and was co-expressed with the MYCN protein in cells of human neuroblastomas (Figure 1D and E) and the neuronal cells of the human cerebrum (Figure 1F and G). It was also co-expressed with the MYCN protein in some primary human cancers, including thyroid cancer (Figure S6). Thus, the NCYM protein is a *de novo* evolved gene product and is endogenously expressed in both normal human tissues and cancers.

### Prognostic significance of *NCYM* expression in human neuroblastoma

**Figure 2:**
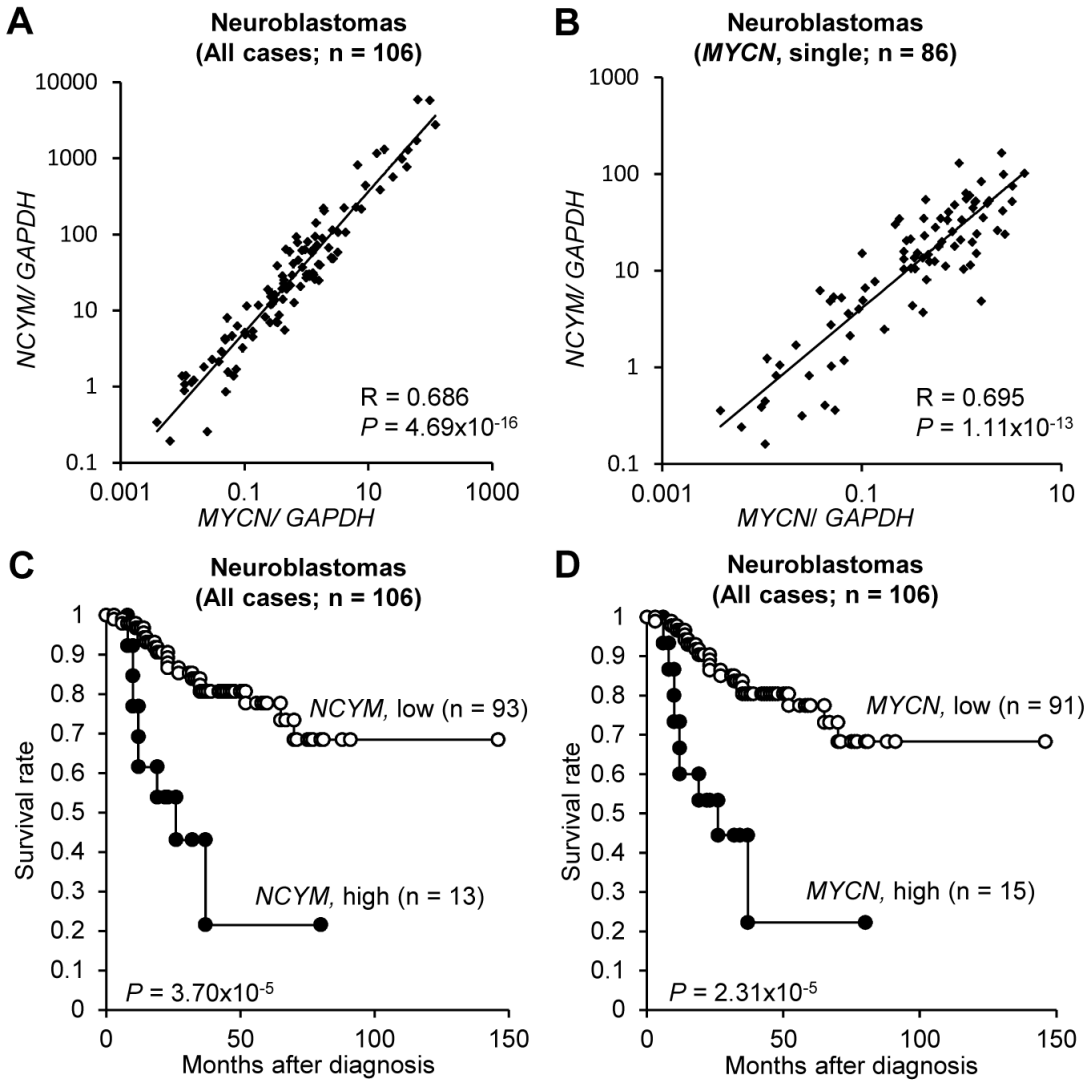
*NCYM* expression is associated with poor prognosis in human neuroblastoma. (A) *NCYM* mRNA expression correlates with that of *MYCN* in human primary neuroblastomas (n = 106, Rs. = 0.686, *P* = 4.69×10^−16^). (B) *NCYM* mRNA expression correlates with that of *MYCN* in human primary neuroblastomas with *MYCN* single copy (n = 86, Rs. = 0.695, *P* = 1.11×10^−13^). The mRNA expression of *NCYM* and *MYCN* was detected by qRT-PCR and normalized using *GAPDH*. (C) Kaplan–Meier survival curves (n = 106, *P* = 3.70×10^−5^, log-rank test). The expression levels of *NCYM* were designated high (n = 13, closed circle) or low (n = 93, open circle) based on the respective average expression. (D) Kaplan–Meier survival curves. The expression levels of *MYCN* were designated high (n = 15, closed circle) or low (n = 91, open circle) based on the respective average expression. High *MYCN* mRNA expression was significantly correlated with poor prognosis (n = 106, *P* = 2.31×10^−5^, log-rank test).

We next examined the prognostic significance of *NCYM* mRNA expression in human neuroblastoma. The *NCYM* gene was co-amplified with the *MYCN* gene in all the cell lines and primary neuroblastomas we examined (Figure S7). *NCYM* expression levels were significantly correlated with that of *MYCN* in primary neuroblastomas (n = 106, *P* = 4.69×10^−16^; Figure 2A) and in the tumors with a single copy of *MYCN* (n = 86, *P* = 1.11×10^−13^; Figure 2B). In addition, high levels of *NCYM* mRNA expression were significantly associated with unfavorable prognostic factors (*P*<0.05, Table S1) and a poor outcome (*P* = 3.70×10^−5^; Figure 2C), similar to that for *MYCN* mRNA expression (*P*<0.05; Table S1 and *P* = 2.31×10^−5^; Figure 2D). Interestingly, high levels of *NCYM* mRNA expression were also significantly correlated with poor outcome in those patients diagnosed at over one year of age without *MYCN* amplification (n = 45, *P* = 0.0375; Figure S8A) whereas those of *MYCN* did not correlate with the prognoses (n = 45, *P* = 0.144; Figure S8B). Multivariate analysis of 106 primary neuroblastomas showed, as expected, that *NCYM* mRNA expression is not an independent prognostic factor from expression and amplification of *MYCN* (Table S2). However, it is an independent prognostic factor from age at diagnosis, stage and *TrkA* expression.

### Positive feedback regulation between NCYM and MYCN

**Figure 3:**
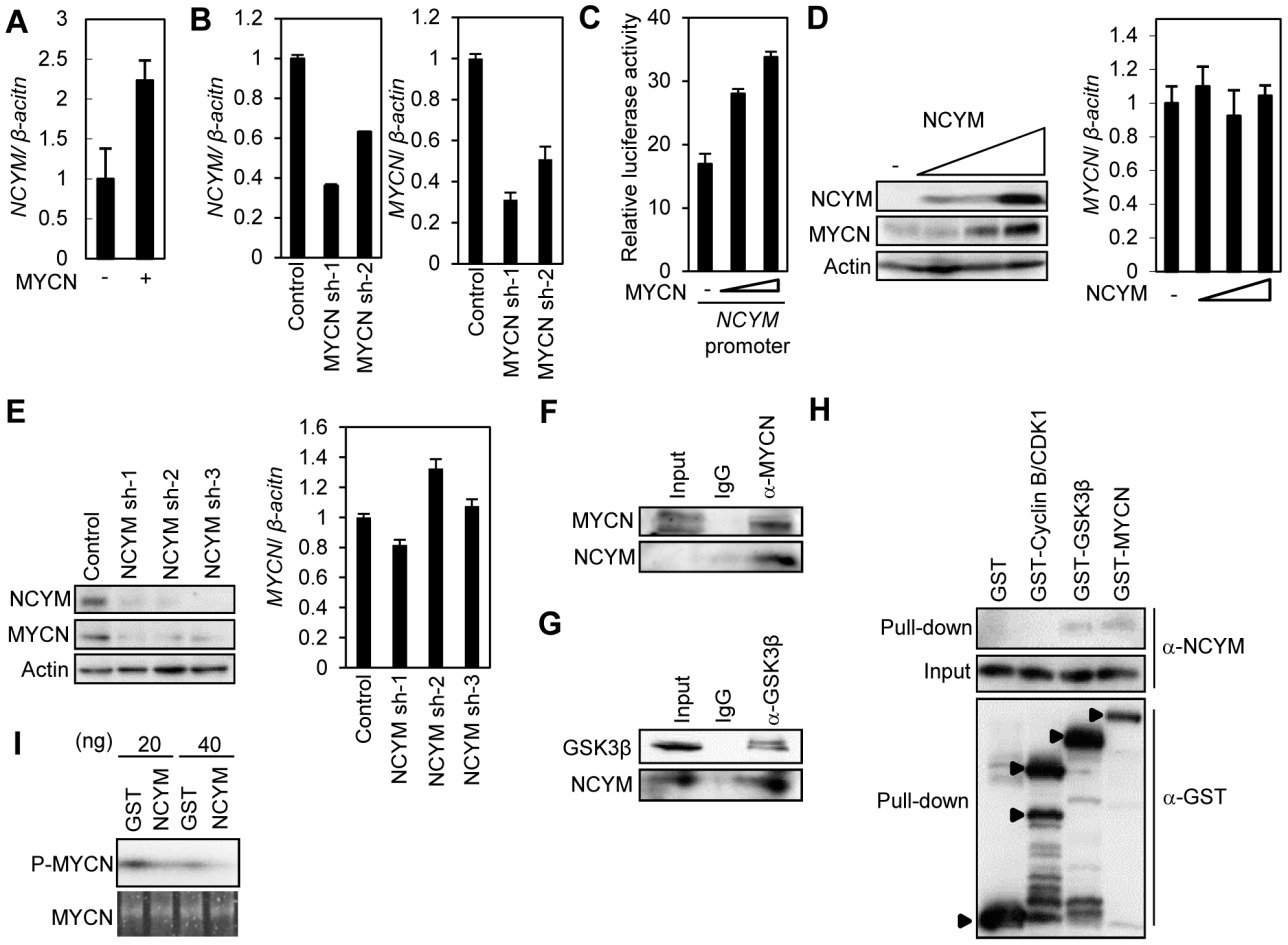
Functional interaction between NCYM and MYCN. (A) Relative mRNA levels of *NCYM* in SK-N-AS *MYCN* single copy human neuroblastoma cells transfected with MYCN expression vector. mRNA levels were measured by qRT-PCR with *β-actin* as an internal control. (B) Relative mRNA levels of *NCYM* (left panel) or *MYCN* (right panel) upon depletion of MYCN in CHP134 human *MYCN*-amplified neuroblastoma cells. (C) MYCN enhances *NCYM* promoter activity. Human neuroblastoma SK-N-AS cells were transfected with increasing amounts of MYCN expression plasmid (0, 200, 300 ng) and their luciferase activity was measured. (D) Western blots showing NCYM overexpression induces MYCN protein in Neuro 2a mouse neuroblastoma cells (left panel). *MYCN* mRNA expression in mouse neuroblastoma Neuro 2a cells transfected with increasing amounts of NCYM expression vector measured by qRT-PCR (right panel). (E) Western blots showing NCYM knockdown decreases MYCN protein in CHP134 cells (left panel). *MYCN* mRNA expression in NCYM knockdown CHP134 cells as measured by qRT-PCR (right panel). (F, G) Co-immunoprecipitation of endogenous NCYM with endogenous MYCN and GSK3β. (H) GST-pulldown assay. Purified NCYM proteins were pulled down with GST-fused GSK3β and MYCN. (I) *In vitro* kinase assay. Radiolabeled ATP was used for the second reaction with GSK3β together with the indicated amount of NCYM or GST. The amount of phosphorylated MYCN was quantified using standard autoradiography. The total amount of the MYCN was quantified by using an Oriole Fluorescent Gel stain.

The co-amplification and co-expression of *NCYM* and *MYCN* in human primary neuroblastomas prompted us to investigate the functional interaction between NCYM and MYCN. Previously we have reported that MYCN directly targets its own expression in neuroblastoma cell lines [27]. Because the promoter region of the *NCYM* gene is localized within intron 1 of *MYCN* (Figure S9A), we examined whether MYCN regulates *NCYM* transcription. Overexpression of MYCN in human neuroblastoma cells induced *NCYM* mRNA expression (Figure 3A), whereas shRNA-mediated knockdown of *MYCN* downregulated endogenous *NCYM* mRNA levels (Figure 3B). MYC overexpression did not induce either *MYCN* or *NCYM* expression (Figure S9B). However, MYCN overexpression did enhance *NCYM* promoter activity in a dose-dependent manner (Figure 3C), suggesting that MYCN, but not MYC, activates the transcription of *NCYM*. Putative E-boxes exist in intron 1 of the *MYCN* gene; however, it is unclear whether they are responsible for this feedback regulation. We therefore generated constructs containing different lengths of the MYCN intron 1 region and performed luciferase assays to identify the MYCN-responsive region (Figure S9C). MYCN enhances its own promoter activity in a dose-dependent manner when co-transfected with reporter plasmids containing the NCYM promoter region (from +1073 to +1312). However, when co-transfected with plasmids without this NCYM promoter region, MYCN positive autoregulation was diminished. Within this region, there is a putative E-box located just 2 base pairs upstream from the transcription start site of the *NCYM* gene (Figure S10A). We generated constructs containing the NCYM promoter region comprising either a wild-type or a mutant E-box. Overexpression of MYCN enhanced NCYM wild-type promoter activity, but mutation of the E-box diminished its activation (Figure S10C). MYC overexpression did not activate either of the NCYM promoter constructs (Figure S10B and C). Therefore, these results indicate that MYCN enhances NCYM promoter activity in an E-box-dependent manner. MYC, however, is not involved in *NCYM* transcription.

We next investigated the function of NCYM in neuroblastoma cells. NCYM overexpression induced MYCN protein levels (Figure 3D, left panel; Figure S11A), but had no effect on the mRNA levels of *MYCN* (Figure 3D, right panel; Figure S11A). Consistent with these results, shRNA-mediated knockdown of NCYM significantly downregulated the amount of MYCN protein without affecting the level of *MYCN* mRNA expression (Figure 3E). In addition, knockdown of NCYM decreased the stability of the MYCN protein (Figure S11B). This NCYM knockdown-mediated destabilization of MYCN could be inhibited using the proteasome inhibitor MG132 (Figure S11C). It is known that the stability of the MYCN protein is regulated by a series of phosphorylation and ubiquitination events that are required for its recognition by the proteasome [28]. CDK1/Cyclin B1 phosphorylates MYCN at serine 62: the mono-phosphorylated MYCN is then recognized by GSK3β and subsequently phosphorylated at threonine 58, leading to its proteasome-dependent protein degradation after an E3-mediated polyubiquitination [28], [29]. Therefore, using immunoprecipitation, we next searched for factors interacting with NCYM that are able to induce MYCN stabilization, and found that NCYM forms a complex with MYCN and GSK3β in CHP134 cells (Figure 3F and G). In addition, purified NCYM was capable of interacting with purified GSK3β and MYCN *in vitro* (Figure 3H). To examine the effect of NCYM on GSK3β-mediated phosphorylation of MYCN, we performed an *in vitro* kinase assay (Figure 3I). NCYM protein inhibited the phosphorylation of MYCN. Because the purified NCYM protein is not a substrate of GSK3β (Figure S12), it is unlikely that NCYM competes with MYCN for GSK3β as a substrate. Taken together these results suggest that the NCYM protein inhibits GSK3β-mediated MYCN phosphorylation and stabilizes the MYCN protein *in vitro*.

It has been reported that MYCN knockdown decreases cell proliferation and induces apoptosis and/or differentiation in *MYCN*-amplified neuroblastoma cells [30]. Therefore, we next investigated the functional role of NCYM in these cells (Figure S13 and S14). We performed NCYM knockdown in BE (2)-C, CHP134, SK-N-AS and SH-SY5Y human neuroblastoma cells. SK-N-AS and SH-SY5Y cells are *MYCN*-single copy but have a high expression of *MYC*, while BE (2)-C and CHP134 are cell lines with *MYCN*-amplification and hence have a high expression of *MYCN* and *NCYM* (Figure S13A). NCYM knockdown did not affect the survival of the *MYCN*-single neuroblastoma cell lines, but promoted massive apoptosis of the *MYCN*-amplified neuroblastoma cells (Figure S13B and C). In addition, in BE (2)-C cells, NCYM knockdown was found to inhibit cell proliferation and invasion (Figure S14B and D). These results suggest that NCYM promotes the survival and aggressiveness of *MYCN*-amplified neuroblastoma cells.

### Co-expression of *MYCN*/*NCYM* in mice promotes neuroblastoma metastasis

**Figure 4:**
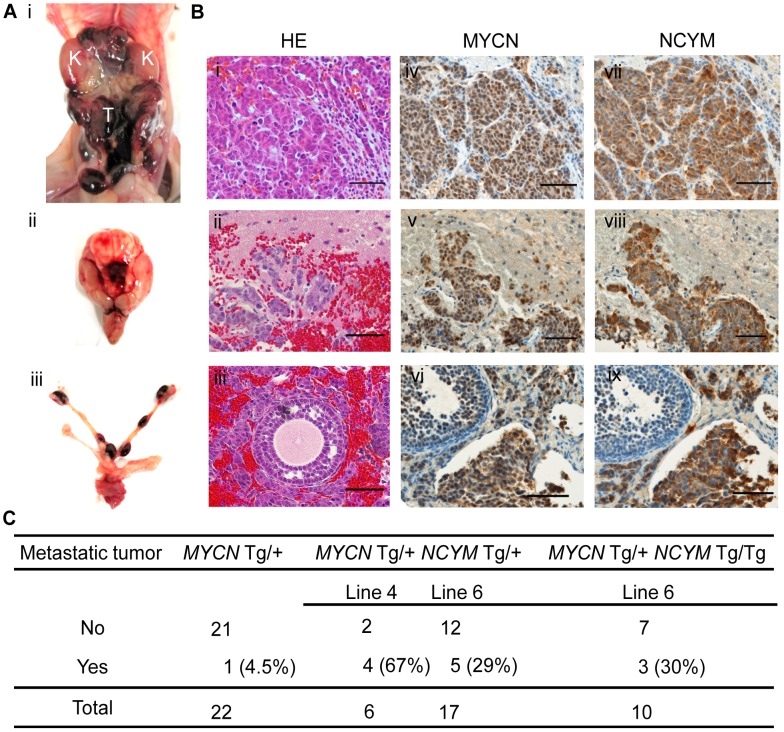
NCYM promotes metastasis in mouse transgenic models of neuroblastoma. (A) Neuroblastomas arise as multifocal primary lesions in a *MYCN*/*NCYM* double transgenic mouse (line 6). (i) Abdominal primary tumors and metastatic tumors in the intracranium (ii) and ovary (iii) occurred within the same mouse (M1). (B) H&E staining (i, ii, iii) and immunohistochemistry for MYCN (iv, v, vi) and NCYM (vii, viii, ix) expression in abdominal tumors (i, iv, vii) and metastatic tumors in the intracranium (ii, v, viii) and ovary (iii, vi, ix), in the MYCN/NCYM transgenic mouse (M1). Scale bars, 50 µm. (C) The rates of metastatic tumor development in *MYCN* and *MYCN*/*NCYM* transgenic mice. Line 6; *P* = 0.036, Mann–Whitney U test. Line 4; *P*<0.01, Fisher's exact probability test.

**Figure 5:**
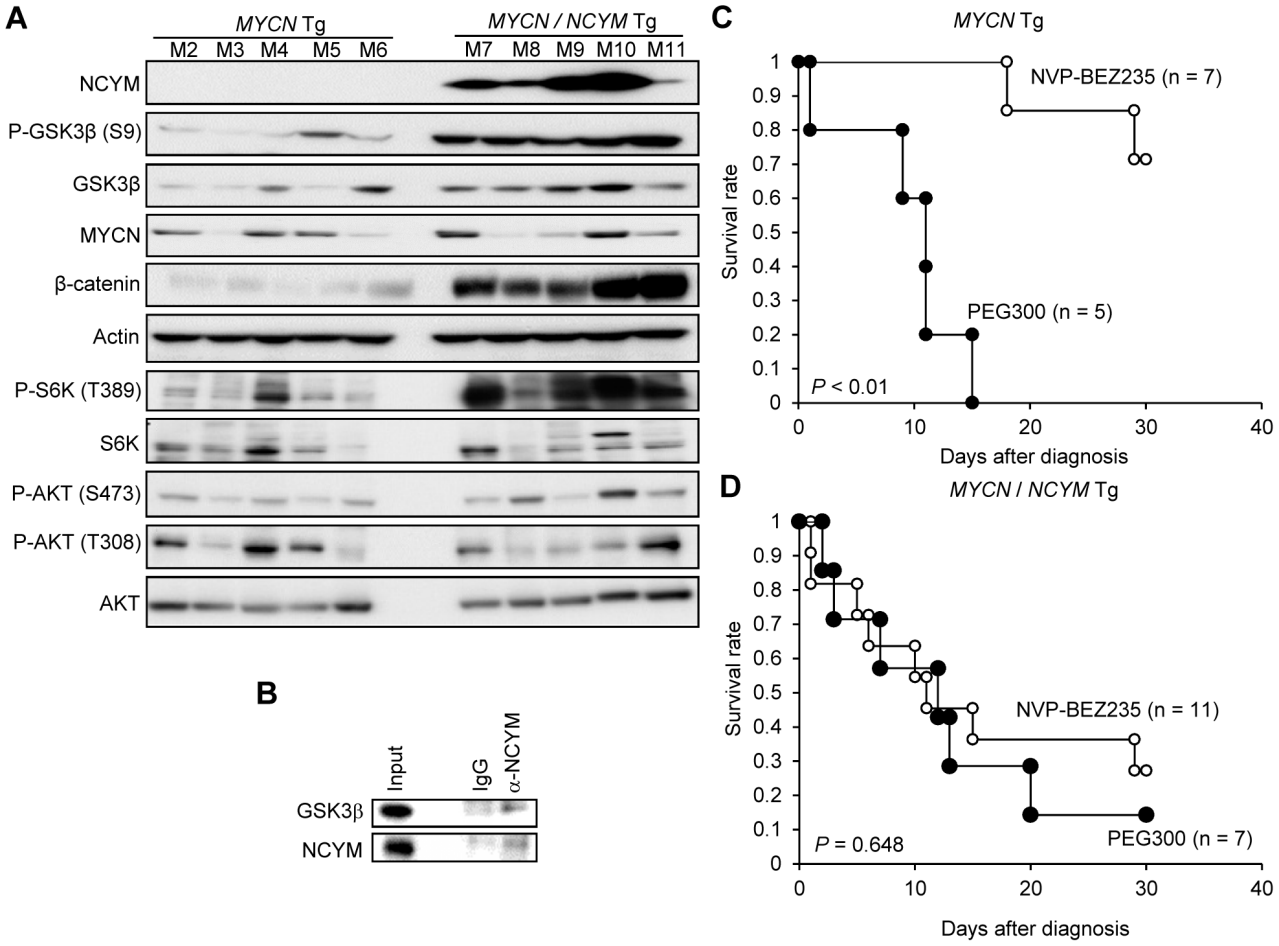
*MYCN*/*NCYM* tumors show drug resistance to a PI3K/mTOR-dual inhibitor. (A) Western blots of *MYCN* (M2–M6) and *MYCN*/*NCYM* (M7–M11) mouse tumors for NCYM, phospho-GSK3β (S9), GSK3β, β-catenin, and MYCN, phospho-S6K (T389), S6K, phospho-AKT (S473), phospho-AKT (T308), and AKT. Actin was used as loading control. (B) NCYM binds to GSK3β *in vivo*. Tumors developed in *MYCN/NCYM* transgenic mice (M12) were immunoprecipitated with control IgG or NCYM antibodies. GSK3β was co-immunoprecipitated with a NCYM antibody. (C, D) Kaplan–Meier survival analysis of *MYCN* mice (panel C, *P*<0.01, log-rank test) or *MYCN*/*NCYM* mice (panel D, *P* = 0.648, log-rank test). Treatment with NVP-BEZ235 (35 mg/kg; open circles) or vehicle (PEG300; closed circles). NVP-BEZ235, *MYCN* transgenic mice n = 7, *MYCN*/*NCYM* transgenic mice n = 11; PEG300, *MYCN* transgenic mice n = 5, *MYCN*/*NCYM* transgenic mice n = 7.

To assess the function of NCYM *in vivo*, we generated transgenic mice expressing the human *NCYM* gene under the control of the rat *tyrosine hydroxylase* (*TH*) promoter (Figure S15A and B). In addition, we made double transgenic mice carrying both the human *MYCN* and *NCYM* genes. *NCYM* Tg/+ mice were mated with *MYCN* Tg/+ *NCYM* Tg/+ mice, and 83 descendants were observed for 200 days (Figure S15C and D). None of the *NCYM* transgenic mice of the 129^+ter^/SVJ background developed neuroblastoma (Figure S15D), suggesting that NCYM overexpression alone is not sufficient to initiate neuroblastoma *in vivo*. Although tumor formation was not accelerated in the *MYCN*/*NCYM* double transgenic mice (Figure S15E), the incidence of neuroblastomas with distant metastases was significantly increased in the *MYCN*/*NCYM* double transgenic mice (Figure 4, Figure S16, Table S3). The overexpression of the MYCN and NCYM proteins in primary and metastatic tumor cells was confirmed by immunohistochemistry (Figure 4B). In the neuroblastoma tissue of the double transgenic mice, GSK3β was significantly inactivated by phosphorylation at serine 9 (Figure 5A). To investigate the mechanism by which NCYM promotes the phosphorylation of GSK3β, we analyzed the phosphorylation status of the known upstream kinases for GSK3β, AKT [28] and S6K [31]. S6K was highly phosphorylated in the *MYCN*/*NCYM* double transgenic mice, whereas AKT was not noticeably activated. The phosphorylation levels of S6K in neuroblastomas from the *MYCN*/*NCYM* double transgenic mice were correlated with the expression levels of MYCN and NCYM (Figure 5A, M7-M11). These results suggest that NCYM promotes the phosphorylation of GSK3β via the activation of mTOR-S6K signaling. Furthermore, NCYM co-immunoprecipitated with GSK3β (Figure 5B) and substrates of GSK3β such as MYCN and β-catenin were stabilized in the neuroblastoma tissues induced in *MYCN*/*NCYM* transgenic mice (Figure 5A). We next examined the number of apoptotic cells in neuroblastomas from *MYCN* transgenic mice and *MYCN*/*NCYM* double transgenic mice by staining for cleaved caspase-3 (Figure S17). The number of apoptotic tumor cells was significantly decreased in the primary tumors of *MYCN*/*NCYM* double transgenic mice, suggesting that NCYM promotes the survival of neuroblastoma cells *in vivo*.

### The tumors which develop in *MYCN*/*NCYM* transgenic mice are resistant to PI3K/mTOR inhibition

To examine whether the overexpression of NCYM contributes to the chemosensitivity of neuroblastomas via GSK3β inhibition, we tested the effect of NVP-BEZ235 on the survival of the *MYCN*/*NCYM* double transgenic mice. NVP-BEZ235 is a dual inhibitor of both PI3K and mTOR and promotes the degradation of MYCN to effectively reduce tumor burden in the *MYCN* transgenic mouse via GSK3β activation [32]. As reported, NVP-BEZ235 treatment significantly prolonged the survival duration of the *MYCN* transgenic mice (*P*<0.01; Figure 5C). In contrast NVP-BEZ235 did not prolong the survival of the *MYCN*/*NCYM* double transgenic mice (*P* = 0.648; Figure 5D). Thus, the expression of NCYM reduced the efficiency of this drug *in vivo*.

## Discussion

Our results reveal that *NCYM*, which was initially thought to be a large non-coding RNA transcribed from a *cis*-antisense gene of human *MYCN* [26], is actually translated into a functional protein in humans. *MYCN* is a highly conserved, major oncogene in human cancer. The newly evolved *cis*-antisense *NCYM* gene product targets the sense *MYCN* gene product, influencing its stabilization, which in turn enhances transcription of the *NCYM* gene. This positive autoregulatory loop may function in primary human neuroblastomas to enhance metastasis as well as drug resistance through stabilization of MYCN and even β-catenin, which are mediated by inhibition of GSK3β (Figure S18). Thus, NCYM is the first *de novo* evolved gene product shown to function in the development of human neuroblastoma.

NCYM promoted phosphorylation of GSK3β at serine 9 possibly via the activation of mTOR-S6K signaling, that might have led to the constitutive inactivation of GSK3β *in vivo*. Recently, Schramm *et al.* reported that MYCN transcriptionally regulates the mTOR pathway, promoting its activation [33]. Thus, MYCN might have enhanced S6K phosphorylation by activating the mTOR pathway in neuroblastomas caused in the double transgenic mice. Previous reports have suggested that neuroblastoma cell lines expressing high levels of MYCN were significantly more sensitive to mTOR inhibitors compared with cell lines expressing low MYCN levels [34]. Furthermore, our study showed that NCYM knockdown significantly induces apoptosis in *MYCN*-amplified neuroblastoma cells, whereas the effects were marginal in *MYCN*-single neuroblastoma cells. Therefore, the feedback regulation between mTOR-S6K signaling and MYCN/NCYM may contribute to the survival of *MYCN*-amplified neuroblastoma cells (Figure S18).

Although NCYM inhibits GSK3β-mediated MYCN phosphorylation *in vitro*, our data does not rule out the possibility that NCYM may stabilize MYCN in a GSK3β-independent manner. Because NCYM binds directly to MYCN both *in vitro* and in neuroblastoma cells, this may affect the recruitment of the regulators of MYCN stability. Indeed, we have recently found that the tumor suppressor protein Runx3 directly binds to MYCN in neuroblastoma cells and promotes degradation of MYCN in the ubiquitin–proteasome system dependent manner [35]. Therefore, the binding of NCYM to MYCN itself could affect the interaction of Runx3, or other regulators such as Aurora A [36] with MYCN to induce its stability. Further studies are required to evaluate the role of NCYM-mediated inhibition of GSK3β activity on MYCN stability.

Recent reports have suggested that both mutant ALK [37], [38] and Lin28B [39] promote the growth of neuroblastomas in transgenic mouse models by targeting MYCN for stabilization [37], [38] or overexpression [39]. Among the known regulators of MYCN, *NCYM* is the only gene that shows 100% co-amplification with *MYCN* in human primary neuroblastomas. Overexpressed NCYM stabilizes both MYCN and β-catenin, and enhances the generation of neuroblastomas with increased aggressive behavior such as distant metastasis and/or drug resistance, which are characteristics reminiscent of human neuroblastoma. Recently, Valentijn *et al.* suggested that the activation of the MYCN pathway is a more significant prognostic factor than the expression or amplification of MYCN in primary neuroblastomas [40]. Consistent with this, our results indicate that *NCYM* expression is associated with poor outcomes in human neuroblastoma regardless of genomic status of the *MYCN*/*NCYM* locus. Therefore, we anticipate that the positive auto-regulatory loop formed by MYCN and NCYM may be a promising target for developing novel therapeutic tools against high-risk neuroblastoma.

As suggested in the recent report [37], the concomitant inhibition of apoptosis and/or activation of survival signals may be required for MYCN to induce multiple tumors or metastases *in vivo*. In this study, we found that NCYM maintains the survival of *MYCN*-amplified neuroblastoma cells, and that the apoptotic cell number, indicated by cleaved caspase-3, was downregulated in *MYCN/NCYM* transgenic mice. In addition, GSK3β inhibition contributes to the inhibition of apoptosis in response to treatment with DNA-damaging drugs in neuroblastoma cells [41]. Therefore, the concomitant activation of other GSK3β substrates, such as β-catenin, and mTOR-S6K signaling by NCYM may be involved in the inhibition of apoptosis (Figure S18).

Since the proposals of Ohno and Jacob, the birth of a new gene has been believed to be caused by the duplication or rearrangement of pre-existing genes [1], [2]. The recent advances in whole genome sequencing technology and bioinformatics have identified the presence of *de novo* proteins; however, their physiological or pathological significance have largely remained unclear [3], [15]. In 2010, Li *et al.* reported that MDF1 originated *de novo* from a DNA sequence previously thought to be non-coding in *Saccharomyces cerevisiae* [7]. MDF1 inhibits mating efficiency by binding MATα2 and promoting vegetative growth. Therefore, while MDF1 was the first reported *de novo* gene whose protein product function was unveiled in a monad, NCYM may be the first *de novo* protein whose precise function has been clarified in multicellular organisms, specifically in humans.

In conclusion, NCYM is a *de novo* evolved protein which acts as an oncopromoting factor in human neuroblastoma. Our results suggest that *de novo* evolved new gene products may be involved in the functional regulation of human cancers and even other diseases.

## Materials and Methods

### Evolutionary analyses

DNA sequences of all species were extracted from the UCSC genome browser on the basis of conservation. From the protein-coding regions, we took the conserved block that was annotated as the region corresponding to the *NCYM* coding sequence, located in exon 3. For intron sequences, we used BLAT [42] to align the *NCYM* mRNA sequence (NR_026766) to the genome of each species and extracted the unmapped regions in the alignment. We found exactly two unmapped regions for each species except for mouse (and thus did not use the mouse sequence). For intergenic regions, we used multiz [43] alignment across 48 species in the browser and cut out 1000-bp sequences that corresponded to human intergenic regions. The sequences of common ancestors were estimated based on the maximum parsimony principle that led to the minimum number of nucleotide-base changes along the already-known phylogenetic tree of the five primates and mice [16]. For multiple possibilities with the same minimum number, we broke the tie by selecting the nucleotide base of the closest outgroup (*e.g.*, when we had A for human, T for chimpanzee, and T for orangutan, we chose T for the common ancestor of human and chimpanzee). When multiple possibilities still remained (as in common ancestor 1), we considered all the possibilities to be equally probable. We estimated common ancestor sequences only within close species (human, chimpanzee, orangutan, and rhesus macaque). We used BLAST [44] to make an alignment between two translated amino-acid sequences ending at the first terminal codon, and calculated *K*_a_ and *K*_s_ using the KaKs_Calculator [45] with the ‘gMYN’ method, where *K*_a_ and *K*_s_ are the rates of non-synonymous and synonymous amino-acid changes, respectively. All pairs of sequences were aligned entirely from the start codon to the terminal codon and did not include any indels, except for the alignment between common ancestor 2 and common ancestor 3, for which we noted ‘frameshift’ instead of the *K*_a_ and *K*_s_ values.

We measured a bias in the codon frequencies (or amino acid frequencies) through the deviation from the uniform usage of each codon, using the Chi-squared statistic normalized to the number of codons:
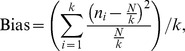
where *n*_1_, …, *n_k_* (*n_i_*≠0) are the observed number of codon 1, …, and that of codon *k*, respectively. *N* is *n*_1_+…+*n_k_*. We used *R* for the calculations and computed the *P-*values using a Monte-Carlo simulation with 10,000 replicates.

### Generation of a human NCYM antibody

A polyclonal anti-NCYM antibody was raised in rabbits against a 14-amino acid stretch at the C-terminal region of NCYM (84-LGTRPLDVSSFKLK-97) (Medical and Biological Laboratories, Nagoya, Japan). The specificity of the purified antibody's affinity was assessed by immunoblotting.

### Immunohistochemistry

Neuroblastoma tissues obtained from mice were fixed in 4% paraformaldehyde and paraffin-embedded for histological studies. Tissue sections were stained with hematoxylin and eosin (H&E) and examined histologically by pathologists for confirmation of the tumor type. Tissue arrays (FDA808a-1 and FDA808a-2, US Biomax, Rockville, MD, USA) were used for the analyses of NCYM or MYCN expression in normal and tumorous human tissues. For immunohistochemistry, tissue sections were stained with the polyclonal anti-NCYM antibody we generated, an anti-MYCN antibody (Calbiochem, San Diego, CA, USA), and cleaved Caspase-3 (Cell Signaling Technology).

### Immunofluorescence

*MYCN*-amplified human neuroblastoma TGW cells grown on coverslips were fixed with 4% paraformaldehyde in 1× PBS for 20 min at 4°C, permeabilized with 0.1% Triton-X for 20 min at room temperature, and then incubated with 2% BSA and 3% goat serum in PBS for 1 h to reduce nonspecific binding. Immunostaining was performed by incubating cells with the polyclonal anti-NCYM antibody and a monoclonal anti-MYCN antibody (Calbiochem) for 2 h at room temperature in a humidified chamber, followed by incubation with fluorescent-conjugated goat anti-rabbit IgG (diluted 1∶400) or fluorescent-conjugated goat anti-mouse IgG (diluted 1∶400), respectively. The coverslips were washed extensively with PBS, mounted in VECTASHIELD mounting medium with DAPI (Vector Laboratories, Burlingame, CA, USA) and images were captured using a confocal microscope (DMI 4000B, Leica).

### Plasmids

We previously made a MYCN-luc (+1312) plasmid that contains the region of MYCN promoter region spanning from −221 to +1312 (where +1 represents the transcription start site) [27]. Luciferase reporter plasmids containing different lengths of the MYCN promoter were generated from MYCN-Luc (+1312) by partial removals of the *MYCN* promoter region with appropriate restriction enzymes. The MYCN promoter region in MYCN-luc (+1312) was subcloned into the pGL3basic vector or pGL4.17Δ*Eco*RV *Eco*RI vector in the opposite direction for generation of NCYM-luc vectors. pGL4.17 Δ*Eco*RV *Eco*RI was the luciferase reporter plasmid, where an *Eco*RV site in pGL4.17 (Promega, Southampton, UK) is replaced with an *Eco*RI site. NCYM-luc E-box WT and NCYM-luc E-box MT were generated by PCR-based amplification using MYCN-luc (+1312) as a template. Oligonucleotide primers used were as follows: 5′-AACCAGGTTCCCCAATCTTC-3′ (forward) and 5′-ACCACCCCCTGCATCTGCAT-3′ (reverse, NCYM-luc E-box WT) or 5′-ACCACCCCCTGCATCCGCAT-3′ (reverse, NCYM-luc E-box MT). Underlined sequences in the reverse primers indicate the wild-type or mutant E-boxes. The NCYM complementary DNA was introduced into a pcDNA3 expression vector, comprising a FLAG-tag at the 5′ locus of NCYM to generate pcDNA3-FLAG-*NCYM*. The sequence of the entire *NCYM* open reading frame was confirmed by sequencing. The FLAG-*NCYM* cDNA was ligated downstream of the rat *TH* promoter in the pGEM7z(f+) expression plasmid, which was originally made from a *MYCN* transgenic construct [21] by excision of the *MYCN* gene, to generate pGEM7z(f+)-FLAG-*NCYM*.

### Cell culture, infection, transfection, and RNA interference

Human neuroblastoma cell lines SH-SY5Y, SK-N-AS, NLF, IMR32, CHP134, and SK-N-BE were maintained in RPMI-1640 medium supplemented with 10% fetal bovine serum (FBS) and antibiotics. Human neuroblastoma cell line BE (2)-C was maintained in a 1∶1 mixture of minimal essential medium (MEM, Gibco by Life technologies, Carlsbad, CA, USA) and Ham's Nutrient Mixture F12 (Gibco) supplemented with 15% heat inactivated fetal bovine serum (FBS) (Gibco) with MEM non-essential amino acids (Gibco) and antibiotics. Mouse neuroblastoma cell line Neuro 2a was maintained in DMEM supplemented with 10% FBS and antibiotics. NLF, IMR32, CHP134, SK-N-BE, and BE (2)-C have amplified *MYCN*, whereas SH-SY5Y, SK-N-AS, and Neuro 2a are cell lines with a single copy of *MYCN*. The cells or tissues with a single copy of *MYCN* have one copy of *MYCN* gene in a haploid genome. Lentivirus was produced by co-transfecting cDNA or shRNA expression plasmids with pCMVR and pMDG plasmids into HEK293T cells using FuGENE HD reagent (Roche, Mannheim, Germany). The MYCN and NCYM shRNA expression plasmids contained pLKO.1-puro as the backbone (Sigma, St Louis, MO, USA). At 24 and 48 h after transfection, the viral supernatants were collected and mixed with neuroblastoma cells. Other plasmid transfections were done using Lipofectamine 2000 transfection reagent (Invitrogen, Karlsruhe, Germany) according to the manufacturer's instructions. The target sequences of the shRNAs used were as follows: NCYM sh-1 (N-cym1 custom shRNA, Sigma) 5′-tggcaattgcttgtcattaaa-3′, NCYM sh-2 (N-cym 2 custom shRNA, Sigma) 5′-gaggttgctcctgtgtaatta-3′, NCYM sh-3 (N-cym 3 custom shRNA, Sigma) 5′-tcctgtgtaattacgaaagaa-3′, MYCN sh-1 (TRCN0000020694, Sigma) 5′-gccagtattagactggaagtt-3′, MYCN sh-2 (TRCN0000020695, Sigma) 5′-cagcagcagttgctaaagaaa-3′. The control shRNA (SHC002) was purchased from Sigma.

### RNA isolation, RT-PCR and quantitative real-time RT-PCR

Total RNA was isolated from the frozen tumor samples and adrenal tissues of transgenic mice with ISOGEN (NIPPON GENE, Tokyo, Japan), and treated with RNase-free DNase I. Total RNA from neuroblastoma cells (CHP134 and SK-N-AS) was prepared using an RNeasy Mini kit (Qiagen, Valencia, CA) following the manufacturer's instruction. cDNA was synthesized using SuperScript II with random primers (Invitrogen). Quantitative real-time RT-PCR (qRT-PCR) using an ABI PRISM 7500 System (Applied Biosystems, Foster City, CA) was carried out using a SYBR green PCR reaction. The primer sets used were as follows: (for clinical experiments using primary neuroblastomas) human *MYCN*, 5′-ggacaccctgagcgattcag-3′, and 5′-aggaggaacgccgcttct-3′, human *NCYM* 5′-ccgacagctcaaacacagaca-3′ and 5′- gtaatggcttctgcgaaaagaaa-3′; (for cellular experiments) human *MYCN*, 5′-tccatgacagcgctaaacgtt-3′ and 5′- ggaacacacaaggtgacttcaaca-3′, human *NCYM*, 5′-cgcccccttaggaacaagac-3′ and 5′- gcgcccctcttctttcaatt-3′, mouse *MYCN*, 5′-tcgggacactaaggagcttca-3′ and 5′-ggaatcttggaccggaacaa-3′, mouse *GAPDH*, 5′-gggaagcccatcaccatct-3′ and 5′-cggcctcaccccatttg-3′. The mRNA levels of each of the genes were standardized by *β-actin* or *GAPDH*.

### Luciferase assay

SK-N-AS cells were co-transfected with the indicated reporter constructs and the pRL-TK *Renilla* luciferase cDNA together with increasing amounts of the expression plasmid for MYCN or MYC. Total DNA per transfection was kept constant (510 ng) by adding pcDNA3 (Invitrogen). Forty-eight hours after transfection, firefly and *Renilla* luciferase activities were measured with a dual-luciferase reporter assay system according to the manufacturer's instructions (Promega).

### Immunoblotting

We resolved cell proteins by SDS-PAGE before electro-blotting onto PVDF membranes. We incubated the membranes with the following primary antibodies overnight: anti-NCYM (1∶1000 dilution), anti-MYCN antibody (1∶1000 dilution; Calbiochem and Cell Signaling), anti-Lamin B (1∶1000 dilution; Calbiochem), anti-α-tubulin (1∶1000 dilution; Santa Cruz, CA, USA), anti-GST (1∶1000; Santa Cruz), anti-GSK3β (1∶1000 dilution; Cell Signaling), anti-phospho-GSK3β (S9) (1∶1000 dilution; Cell Signaling), anti-β-catenin (1∶1000 dilution; Cell Signaling), anti-phospho-AKT (S473) (1∶1000 dilution; Cell Signaling), anti-phospho-AKT (S308) (1∶1000 dilution; Cell Signaling), anti-AKT (1∶1000 dilution; Cell Signaling), anti-S6K (1∶1000 dilution; Cell Signaling), anti-phospho-S6K (T389) (1∶1000 dilution; Cell Signaling), and anti-actin (1∶4000 dilution; Sigma). The membranes were then incubated with a horseradish peroxidase-conjugated secondary antibody (anti-rabbit IgG at 1∶2000–1∶4000 dilution or anti-mouse IgG at 1∶2000 dilution; both from Cell Signaling Technology) and the bound proteins were visualized using a chemiluminescence-based detection kit (ECL and ECL pro kit, Amersham, Piscataway, NJ, USA; ImmunoStar LD, Wako).

### Immunoprecipitation

Whole lysates prepared from CHP134 cells or tumor tissues were pre-cleared by incubation with protein G-Sepharose beads (Amersham Pharmacia Biotech) for 1 h at 4°C. The supernatant was collected after a brief centrifugation, and incubated with the indicated primary antibodies at 4°C overnight. The immune complexes were precipitated with protein G-Sepharose beads for 1 h at 4°C, and the non-specific bound proteins were removed by washing the beads with lysis buffer five times at 4°C. Different lysis buffers were used for the cell-based experiments (50 mM Tris-HCl pH 8.0, 137 mM NaCl, 2.7 mM KCl, and 1% Triton X) and for the tumor tissues (50 mM Tris-HCl pH 8.0, 1 mM EDTA, 0.2% DOC and 0.2% SDS). The immunoprecipitated proteins were eluted by boiling in Laemmli sample buffer and analyzed by immunoblotting.

### Analysis of MYCN stability

CHP134 cells were cultured with 50% lentiviral supernatant for transfection of the indicated shRNA. Forty-eight hours after the transfection, cycloheximide (Sigma) was added to the culture medium at a final concentration of 50 µg/ml and cells were harvested at the indicated time points. For MG132 treatment, 44 h after the transfection, cells were treated with DMSO or 10 µM MG132 for 4 h.

### Purification of NCYM protein from bacteria

DH5α cells were transformed with pGEX-4T-*NCYM* plasmid and cultured in Luria Broth (LB) at 37°C. The expression of the GST-NCYM fusion protein was induced by culturing the cells with 1 mM IPTG for 10 h at 25°C. Cells were collected by centrifugation, dissolved in cell lysis buffer (PBS, 1% TritonX-100, 5 mM EDTA and protease inhibitors), and stored at −80°C. Cell extracts were obtained by thawing the frozen cells, followed by sonication and ultra-centrifugation. After a pulldown with glutathione sepharose 4B beads, the beads were washed five times in cell lysis buffer and once in thrombin buffer containing 50 mM Tris-HCl pH 8.0, 150 mM NaCl, 2.5 mM CaCl_2_, 5 mM MgCl_2_, 1 mM DTT. GST-Tag cleavage mediated by thrombin released the full-length NCYM protein from the beads and the thrombin was removed by adding p-aminobenzamidine agarose beads according to the standard protocol. The full length NCYM protein was further purified by filtration using Amicon Ultra-4 (Millipore, Temecula, CA, USA), and dissolved in stock buffer (50 mM Tris-HCl pH 8.0, 150 mM NaCl, 5 mM EDTA, 0.25 mM DTT, 10% sodium azide, 50% glycerol and protease inhibitors) and stored at −20°C. Complete PIC (Roche) was used for protease inhibition.

### GST-pulldown assay

For GST-pulldown assay, 0.5 µg of purified NCYM proteins were incubated with 0.5 µg of GST protein or GST-fused CDK1/Cyclin B1 (Signal Chem, Richmond, Canada), GSK3β (Promega) and MYCN (Abnova, Taipei, Taiwan) for 2 h at 4°C. Bound complexes were recovered on the glutathione-sepharose beads, washed with the binding buffer (50 mM Tris-HCl, pH 8.0, 1 mM EDTA, 150 mM NaCl, 0.1% Nonidet P-40 and Complete PIC), boiled in in Laemmli sample buffer and analyzed by immunoblotting.

### *In vitro* kinase assay

For MYCN phosphorylation, two kinase reactions were performed sequentially. The first kinase reactions were performed for 1 h in kinase buffer (40 mM Tris-HCl pH 7.5, 20 mM MgCl_2_, 0.1 mg/ml BSA, 50 µM DTT) in the presence of 50 µM Ultrapure ATP (Promega), 50 ng of purified MYCN (Abnova), and 40 ng of purified CDK1/Cyclin B1 (Signal Chem) at room temperature. At 1 h, the first reaction solution was mixed with the same volume of kinase buffer containing 100 nM CDK1 inhibitor (CGP74514A, Calbiochem), 4 µCi of [γ-^32^P] ATP (PerkinElmer), and 20 ng of purified GSK3β with the indicated amounts of purified NCYM or purified GST. The second reaction was performed for 1 h at room temperature. The amount of phosphorylated MYCN was quantified using standard autoradiography. The total amount of MYCN was quantified by using an Oriole Fluorescent Gel stain (Bio-Rad). We also examined whether purified NCYM could be a substrate of GSK3β using the ADP-Glo system (Promega) according to manufacturer's instructions. Reactions were performed for 1 h in kinase buffer (40 mM Tris-HCl pH 7.5, 20 mM MgCl_2_, 0.1 mg/ml BSA, 50 µM DDT) in the presence of 25 µM Ultrapure ATP (Promega) and 25 ng of purified GSK3β with increasing amounts of NCYM or GST at room temperature. The peptide of human muscle glycogen synthase-1 (YRRAAVPPSPSLSRHSSPHQ(pS)EDEEE) was used as a positive control for the GSK3β substrate. At 1 h, the reaction solutions were mixed and incubated with ADP-Glo reagent for 40 min at room temperature, and the mixture was combined with a kinase detection reagent and allowed to stand for 30 min. The kinase activities were detected using a luminometer (PerkinElmer ARVOX3).

### TUNEL staining

The indicated neuroblastoma cells were transfected with the indicated shRNA with 50% lentiviral supernatant. Seventy-two hours after transfection, all cells were collected by centrifugation, attached onto the coverslips by CYTOSPIN 4 (Thermo Fisher Scientific, Wilmington, DE, USA), and fixed in 4% paraformaldehyde for 1 h. Apoptotic cells were detected by using an *in situ* cell death detection kit (Roche) according to the manufacturer's protocol. The coverslips were mounted with DAPI-containing mounting medium (Vector Laboratories) and observed under a confocal microscope.

### Cell viability assay (MTT assay)

Cell viability was quantified by the 3-(4, 5-dimethylthiazol-2-yl)-2, 5-diphenyltetrazolium bromide (MTT) method. Cells were collected and seeded in 96-well plates at 1×10^4^ cells/ml. After addition of 10 µl of MTT tetrazolium salt (Sigma) solution to each well, the plates were incubated in a CO_2_ incubator for 60 min. The absorbance of each well was measured using a Dynatech MR5000 plate reader with a test wavelength of 450 nm and a reference wavelength of 630 nm.

### Migration and invasion assay

The invasive potential of BE (2)-C cells *in vitro* was measured by evaluating the number of invading cells using Matrigel-coated trans-well inserts (BD Biosciences) according to the manufacturer's instructions. BE (2)-C cells transfected with the indicated shRNA were seeded onto an insert containing 8 µm pores (BD Biosciences) in a 24-well plate at 1×10^5^ cells/ml. Cells on the lower side of the membrane were fixed with 4% paraformaldehyde and stained using a Diff Quick Staining Kit (Sysmex).

### Generation of transgenic mice

All animal experimental procedures used in this study were reviewed and approved by the Committee on the Ethics of Animal Experiments of the Chiba Cancer Center (Permit Number: 12–13). Linearized and purified pGEM7z (f+)-FLAG-*NCYM* was injected into the pronuclei of fertilized eggs derived from 129/SvJ×C57BL/6J mice. We selected four lines of *NCYM* transgenic mice according to the level of *NCYM* expression in adrenal tissues (Figure S15B), and the transgenic mice were backcrossed to 129/SvJ at least 10 times to generate *NCYM* transgenic mice. To generate *MYCN*/*NCYM* double transgenic mice, the *NCYM* transgenic mice were crossed with *MYCN* transgenic mice of the 129/SvJ strain. On the basis of breeding schemas, all mice carrying the *MYCN* transgene were hemizygous. Tail DNA was analyzed for *MYCN* and *NCYM* transgenes, and the *NCYM* transgene copy number was quantified by quantitative genomic PCR. The primer sets used for genotyping were as follows: *NCYM*, 5′-cgcccccttaggaacaagac-3′ and 5′- gcgcccctcttctttcaatt-3′, *MYCN*, 5′-tggaaagcttcttattggtagaaacaa-3′ and 5′-agggatcctttccgccccgttcgttttaa-3′.

### Detection of metastatic tumors in mice

If more than one tumor over 2 mm in a diameter separately developed in a different organ, we defined this as the mouse having macroscopic metastatic tumors. In Figure 4C, only the number of mice with macroscopic metastatic tumors was counted. As a preliminary experiment, we used microscopy to detect tumors in the brain, pancreas, spleen, heart, lungs, kidneys and liver in nine mice (*MYCN*/*NCYM* double transgenic mice; n = 6, *MYCN* transgenic mice; n = 3). In addition to macroscopic metastases in the brain, heart, ovary and uterus, we found microscopic metastases in the lungs of *MYCN*/*NCYM* double transgenic mice, but the mass of these tumor cells was not large enough to be visible by eye. We also microscopically analyzed the HE-stained bone marrow from the hind legs of 19 mice (*MYCN*/*NCYM* double transgenic mice, n = 10; *MYCN* transgenic mice, n = 9). However, no metastatic tumor cells were found in the bone marrow.

### Murine therapy

All mice were genotyped to detect the presence of human *MYCN* or *NCYM* transgenes. After weaning, at about 30 days old, *MYCN* transgenic mice or *MYCN*/*NCYM* double transgenic mice were palpated for intra-abdominal tumors every day. Mice of either genotype found with palpable tumors were treated with NVP-BEZ235 (Cayman Chemical, Ann Arbor, MI, USA) (35 mg/kg in PEG300) or vehicle (PEG300, Wako) once daily for 30 days by oral gavage. All mice were monitored until euthanasia was required in accordance with the institutional animal committee.

### Tumor specimens

The 106 human neuroblastoma specimens used in the present study were kindly provided by various institutions and hospitals in Japan to the Chiba Cancer Center Neuroblastoma Tissue Bank. Written informed consent was obtained at each institution or hospital. This study was approved by the Chiba Cancer Center Institutional Review Board. Tumors were classified according to the International Neuroblastoma Staging System (INSS): 27 Stage 1, 15 Stage 2, 34 Stage 3, 23 Stage 4, and 7 Stage 4 s. Clinical information including age at diagnosis, tumor origin, Shimada's histology, prognosis and survival duration of each patient was obtained. The patients were treated following the protocols proposed by the Japanese Infantile Neuroblastoma Cooperative Study and the Group for the Treatment of Advanced Neuroblastoma and subjected to survival analysis. Cytogenetic and molecular biological analysis of all tumors was also performed by assessing DNA ploidy, *MYCN* amplification and *TrkA* expression, as previously described [46].

### Array CGH analysis

Array CGH analysis was conducted using the Human Genome CGH 244K Oligo Microarray Kit (G4411B, Agilent Technologies, Santa Clara, CA, USA). Genomic DNA prepared from primary neuroblastoma tissues or cell lines was labeled with Cy3-dye using a QuickAmp labeling kit. Human placental DNA was labeled with Cy5-dye and used as a reference control. Labeling, hybridization and subsequent data processing by FeatureExtraction and CGH-Analytics software were performed according to the manufacturer's instructions. Relative copy number of the probes surrounding the *MYCN* and *NCYM* genomic locus (from *DDX1* to *FAM49A*) were compared in each primary tumor or cell line.

### Statistical analysis

Statistical significance was tested as follows: two-group comparison of survival by log-rank test, correlation of gene expression by Pearson's correlation coefficient test or Student's *t*-test, multivariate analysis for survival by Cox regression model, and the rate of mouse genotype and metastatic tumor occurrence in line 6 was calculated by Chi-square independence test and Mann–Whitney U test, respectively.

## Supporting Information

Figure S1 Alignment of NCYM coding sequences. Primate sequences were extracted from the UCSC genome browser on the basis of conservation, and common-ancestor sequences were estimated based on the maximum parsimony principle. Nucleotide changes are colored in orange. Post-terminal sequences are colored in blue. Post-terminal sequence refers to the DNA sequence after the first terminal codon up to the position corresponding to the first terminal codon in the human sequence. CA indicates common ancestor.
(TIF)

Figure S2 Analysis of the bias of codon (and amino acid) usage and evolutionary rates in the *NCYM* gene. (A) Distribution of NCYM protein length, numbers of end codons, and the bias of codon and amino acid usage. Asterisk indicates statistical significance (*P*<0.001, Monte-Carlo Chi-square test). Graph showing the number of codons (B) or amino acids (C) in the NCYM protein of different species.
(TIF)

Figure S3 Exogenous NCYM protein can be expressed in cells. (A) Purification of NCYM protein from bacteria. GST fusion NCYM was overexpressed in bacterial cells and purified by GST-pulldown. The GST-NCYM protein was further cleaved by thrombin, and full-length NCYM was purified. The left panel shows CBB staining and the right panel shows a western blot using anti-NCYM antibody. The arrow indicates the NCYM protein; the asterisk indicates the degraded NCYM protein. (B) Human NCYM protein expression in mouse neuroblastoma Neuro 2a cells. Neuro 2a cells were transfected with increasing amounts of NCYM expression plasmid (1, 1.5, 2 µg) for 48 h. The cell lysates were subjected to western blotting to verify the expression of human NCYM using an anti-NCYM antibody. The arrow indicates the NCYM protein; the asterisk indicates a non-specific band.
(TIF)

Figure S4 Subcellular localization of NCYM protein in neuroblastoma cells. (A) Localization of NCYM protein in neuroblastoma cells. The indicated neuroblastoma cells were biochemically fractionated into nuclear and cytoplasmic fractions followed by immunoblotting with anti-NCYM antibody. Lamin B and α-tubulin were used as nuclear and cytoplasmic markers, respectively. SK-N-AS and SH-SY5Y are human neuroblastoma cells with a single copy of *MYCN*, and NLF, IMR32, CHP134, and SK-N-BE are human neuroblastoma cells with amplified *MYCN*. (B) Nuclear staining of NCYM and MYCN protein in *MYCN*-amplified human neuroblastoma TGW cells analyzed by confocal fluorescence microscopy. Scale bar, 50 µm.
(TIF)

Figure S5 NCYM protein expression in human normal and neuroblastoma tissues analyzed by immunohistochemistry. The indicated human normal tissues (tissue array, FDA808a-1) were stained with anti-NCYM antibody. (A) Cerebellum; scale bar, 100 µm. (B) Testis; scale bar, 50 µm. (C) Pancreas; scale bar, 100 µm. (D) Heart; scale bar, 100 µm. (E) Human metastatic neuroblastoma in the liver (Stage 4S); scale bar, 50 µm. (F) Human metastatic neuroblastoma in the lymph node (Stage 4); scale bar, 50 µm.
(TIF)

Figure S6 Expression of NCYM and MYCN protein in human thyroid tumors analyzed by immunohistochemistry. Normal and cancerous human tissues (tissue array, FDA808a-2) were stained with anti-NCYM (A and B) or anti-MYCN antibody (C and D). (A), (C), Normal thyroid. (B), (D), Thyroid tumors. Scale bars, 100 µm.
(TIF)

Figure S7 Co-amplification of *MYCN* and *NCYM* genes in human neuroblastoma cell lines and primary neuroblastomas. Average gene copy number was calculated based on the signals of multiple probes targeted to the indicated gene in array CGH. Twenty-three *MYCN*-amplified human neuroblastoma cell lines (A) or 23 human primary neuroblastomas (B) were analyzed by array CGH.
(TIF)

Figure S8 High *NCYM* mRNA expression is associated with poor prognosis in neuroblastomas without *MYCN* amplification. *MYCN* non-amplified neuroblastomas diagnosed at over one year of age were analyzed using Kaplan–Meier survival curves based on the expression levels of *NCYM* mRNA (A) or *MYCN* mRNA (B). The expression levels of *NCYM* or *MYCN* mRNA were examined by qRT-PCR and normalized by *GAPDH*. The average of the expression levels was used as a threshold to divide the tumors with low expression (open circle; A, n = 36, B, n = 34) from those with high expression (closed circle; A, n = 9, B, n = 11). *P* values of (A) and (B) were 0.0375 and 0.144, respectively (Log-rank test).
(TIF)

Figure S9 MYCN, but not MYC, activates *MYCN* transcription in human neuroblastoma cells. (A) Schematic drawing of the *MYCN*/*NCYM* promoter region. (B) Relative mRNA levels of *MYC*, *MYCN* and *NCYM* in SK-N-AS *MYCN* single copy human neuroblastoma cells transfected with 2 µg of a MYC expression vector. mRNA levels were measured by qRT-PCR with *β-actin* as an internal control. (C) Luciferase reporter assays. SK-N-AS cells were transiently co-transfected with a constant amount of the indicated luciferase reporter constructs bearing various lengths of the human *MYCN* promoter region (100 ng), a *Renilla* luciferase reporter plasmid (pRL-TK) (10 ng), and either an empty plasmid (pcDNA3) or with an increasing amount (200, 300, 400 ng) of the expression plasmid for MYCN. Forty-eight hours after transfection, cells were lysed and their luciferase activities were measured.
(TIF)

Figure S10 An intact upstream E-box is required for the activation of the NCYM promoter by MYCN. (A) The NCYM promoter sequence. The sequences of primer sets used in our previous report [27] are shown as MYCN ChIP Forward (Reverse) Primer. The recruitment of the MYCN protein to its own intron 1 was detected using those primers. The putative E-box sequence is indicated in red characters. (B) MYCN, but not MYC, enhances NCYM promoter activity. Human neuroblastoma SK-N-AS cells were transfected with 400 ng of the MYCN expression plasmid for 48 hours and then their luciferase activity was measured. (C) The effect of an E-box mutation on MYCN-induced NCYM promoter activity. The WT and mutant NCYM promoters were evaluated for transcriptional activity 24 hours after the transfection of the expression plasmids for MYCN or MYC. The asterisks indicate statistical significance (*P*<0.01, Student's *t*-test).
(TIF)

Figure S11 NCYM stabilizes the MYCN protein in the ubiquitin–proteasome system dependent manner. (A) Western blot analysis and RT-PCR. Both NCYM and its SNP type (NCYML70V) induce MYCN expression levels in CHP134 cells. (B) Western blot analysis of MYCN expression in CHP134 cells transfected with NCYM or control shRNA, followed by treatment with 50 µM cycloheximide (CHX), and harvested at the indicated time points. (C) Proteasome inhibitor MG132 treatment of NCYM knockdown CHP134 cells. Western blot analysis showed that NCYM-mediated downregulation of MYCN is inhibited by MG132 treatment. Actin was used as a loading control.
(TIF)

Figure S12 GSK3β does not phosphorylate NCYM protein. (A) *In vitro* kinase assay. The phosphorylation of the control substrate human glucose synthase 1 by GSK3β was measured to test the assay system. (B) The correlation between the percentage of ADP and the relative kinase activity measured by luciferase activity (R^2^ = 0.9994). (C) The relative kinase activity of GSK3β was not increased when GST or NCYM were used as a substrate.
(TIF)

Figure S13 NCYM knockdown promotes apoptosis in *MYCN*-amplified neuroblastoma cells. (A) The relative mRNA levels of *MYCN*, *NCYM* and *MYC* in human neuroblastoma cells. Levels of mRNA were measured by qRT-PCR with *β-actin* as an internal control. (B and C) TUNEL staining. The indicated human neuroblastoma cells were lentivirally transfected with the indicated shRNA. Sevently-two hours after the transfection, cells were fixed in 4% paraformaldehyde and subjected to TUNEL staining. Cell nuclei were stained with DAPI (upper panels). Scale bars, 50 µm. The percentage of TUNEL-positive cells was calculated as the average of three different microscopic fields. SH-SY5Y and SK-N-AS are *MYCN* single copy cells (B), and BE (2)-C and CHP134 are *MYCN*-amplified cells (C).
(TIF)

Figure S14 NCYM knockdown inhibits the proliferation and invasion in BE (2)-C cells. (A) Western blot analysis showed that NCYM knockdown decreased MYCN protein in BE (2)-C cells. (B) Cell proliferation assay. After transfection with NCYM shRNA (closed circle) or control shRNA (open circle), cell proliferation was examined in an MTT assay, at the indicated time points. (C, D) the effect of NCYM knockdown on cellular migration (C) and invasion (D). Three days after the introduction of NCYM shRNA, the cells were adjusted to 1×10^5^ cells/ml and subjected to a Boyden chamber invasion assay.
(TIF)

Figure S15 Generation of *MYCN/NCYM* transgenic mice. (A) A *NCYM* cDNA was ligated 3′ to the rat *TH* promoter to generate the pGEM7z(f+)-FLAG-*NCYM* transgenic constructs. (B) *NCYM* mRNA expression in the adrenal tissues of *NCYM* transgenic mice was measured by qRT-PCR. The expression levels were normalized to mouse *GAPDH*. Red arrows indicate the mouse lines used for further experiments. (C) Table showing the distribution of actual numbers of transgenic mice (line 6) resulting from intercrossing of *MYCN* Tg/+ *NCYM* Tg/+ and *NCYM* Tg/+ and the corresponding theoretical numbers (*P*>0.05, Chi-square independence test). This result indicates that the *NCYM* and *MYCN* transgenes have a marginal effect on the embryonic lethality of mice. (D) Kaplan–Meier survival curves of 83 mice resulting from intercrosses of *MYCN* Tg/+ *NCYM* Tg/+ and *NCYM* Tg/+ (mouse line 6). (E) Kaplan–Meier analysis for tumor incidences in *MYCN* Tg/+ and *MYCN*/*NCYM* Tg mice (mouse line 6).
(TIF)

Figure S16 Neuroblastoma histology of *MYCN* transgenic mice and *MYCN/NCYM* double transgenic mice. (A) Neuroblastomas arise as primary lesions in a *MYCN*/*NCYM* double transgenic mouse (i) and *MYCN* transgenic mice (ii). Thoracic paraspinal (T1) and abdominal (T2, T3) tumors. K, kidney. (B) H&E staining of T1 (i), T2 (ii), and T3 (iii).
(TIF)

Figure S17 NCYM inhibits apoptosis in the neuroblastomas of *MYCN*/*NCYM* double transgenic mice. The number of apoptotic cells in neuroblastomas from *MYCN* transgenic mice (A) and *MYCN*/*NCYM* double transgenic mice (B) were measured using cleaved capase-3 staining. Scale bar, 50 µm. (C) Quantification of cleaved caspase-3–positive areas in the tumors. The apoptotic cells were calculated by averaging the number of cleaved caspase-3–positive areas counted in 5 randomly selected fields (100 µm^2^) per slide using WinROOF software (version 7.0, Mitani Corp.). *P* value was 0.012 (Student's *t*-test).
(TIF)

Figure S18 Schematic model of NCYM function in aggressive human neuroblastomas.
(TIF)

Table S1 Correlation between the expression of *NCYM* or *MYCN* and other prognostic factors.
(DOC)

Table S2 Multiple Cox regression analyses of *NCYM* expression, *MYCN* expression, age, *MYCN* amplification, stage, DNA index, Shimada pathology, *TrkA* expression, and origin.
(DOC)

Table S3 Summary of mice bearing neuroblastomas.
(DOC)
