# Supplementary figures and images for: NCYM, a Cis-Antisense Gene of MYCN, Encodes a De Novo Evolved Protein That Inhibits GSK3β Resulting in the Stabilization of MYCN in Human Neuroblastomas
Source: PLoS Genet. 2014 Jan 2;10(1):e1003996. doi: 10.1371/journal.pgen.1003996 (PMC3879166; doi:10.1371/journal.pgen.1003996)

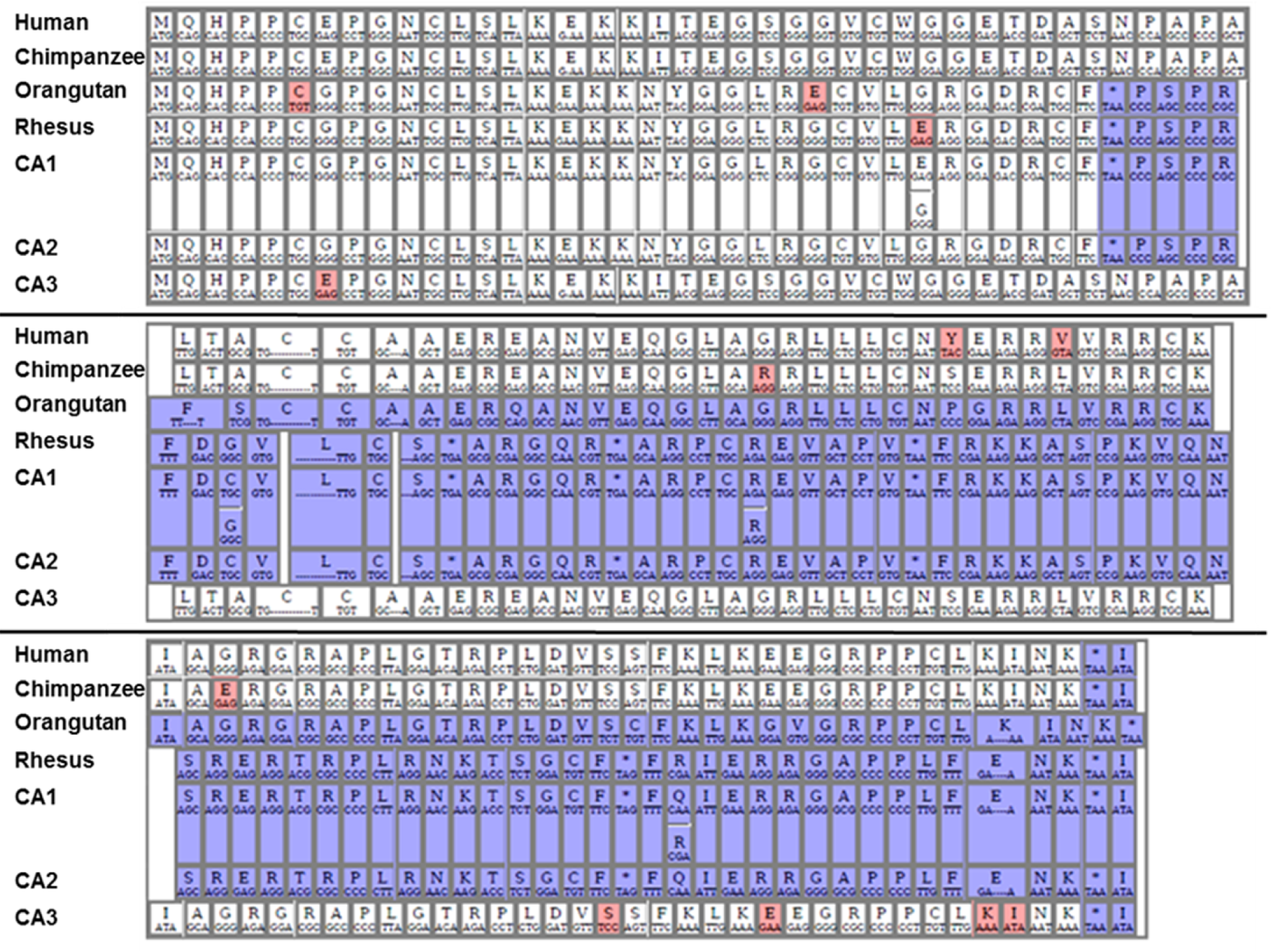

Supplement: Figure S1 — Alignment of NCYM coding sequences. Primate sequences were extracted from the UCSC genome browser on the basis of conservation, and common-ancestor sequences were estimated based on the maximum parsimony principle. Nucleotide changes are colored in orange. Post-terminal sequences are colored in blue. Post-terminal sequence refers to the DNA sequence after the first terminal codon up to the position corresponding to the first terminal codon in the human sequence. CA indicates common ancestor. (TIF) [file pgen.1003996.s001.tif]

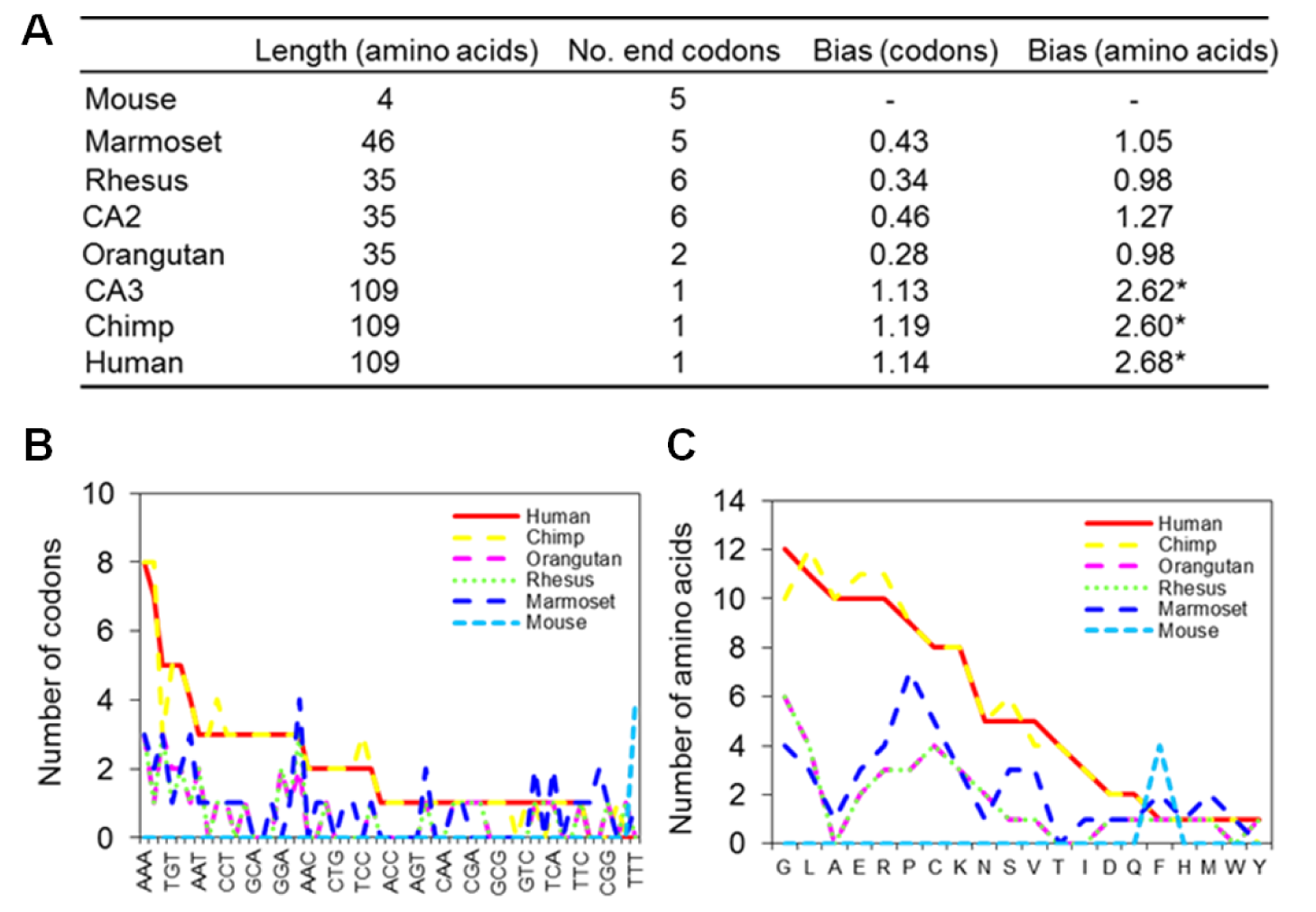

Supplement: Figure S2 — Analysis of the bias of codon (and amino acid) usage and evolutionary rates in the NCYM gene. (A) Distribution of NCYM protein length, numbers of end codons, and the bias of codon and amino acid usage. Asterisk indicates statistical significance (P<0.001, Monte-Carlo Chi-square test). Graph showing the number of codons (B) or amino acids (C) in the NCYM protein of different species. (TIF) [file pgen.1003996.s002.tif]

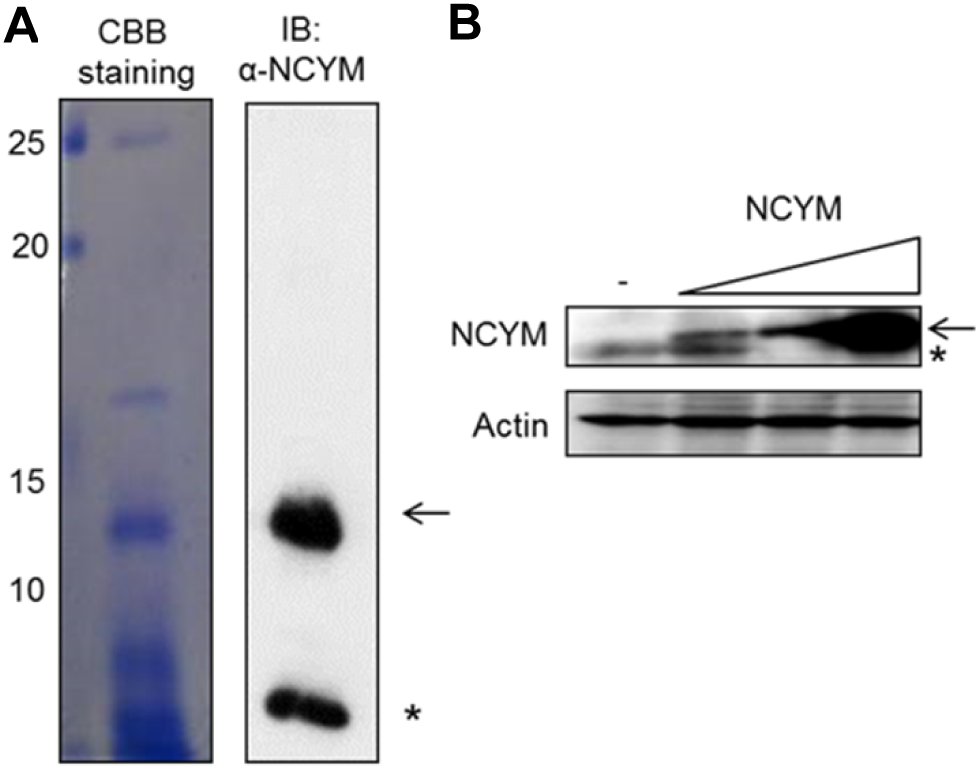

Supplement: Figure S3 — Exogenous NCYM protein can be expressed in cells. (A) Purification of NCYM protein from bacteria. GST fusion NCYM was overexpressed in bacterial cells and purified by GST-pulldown. The GST-NCYM protein was further cleaved by thrombin, and full-length NCYM was purified. The left panel shows CBB staining and the right panel shows a western blot using anti-NCYM antibody. The arrow indicates the NCYM protein; the asterisk indicates the degraded NCYM protein. (B) Human NCYM protein expression in mouse neuroblastoma Neuro 2a cells. Neuro 2a cells were transfected with increasing amounts of NCYM expression plasmid (1, 1.5, 2 µg) for 48 h. The cell lysates were subjected to western blotting to verify the expression of human NCYM using an anti-NCYM antibody. The arrow indicates the NCYM protein; the asterisk indicates a non-specific band. (TIF) [file pgen.1003996.s003.tif]

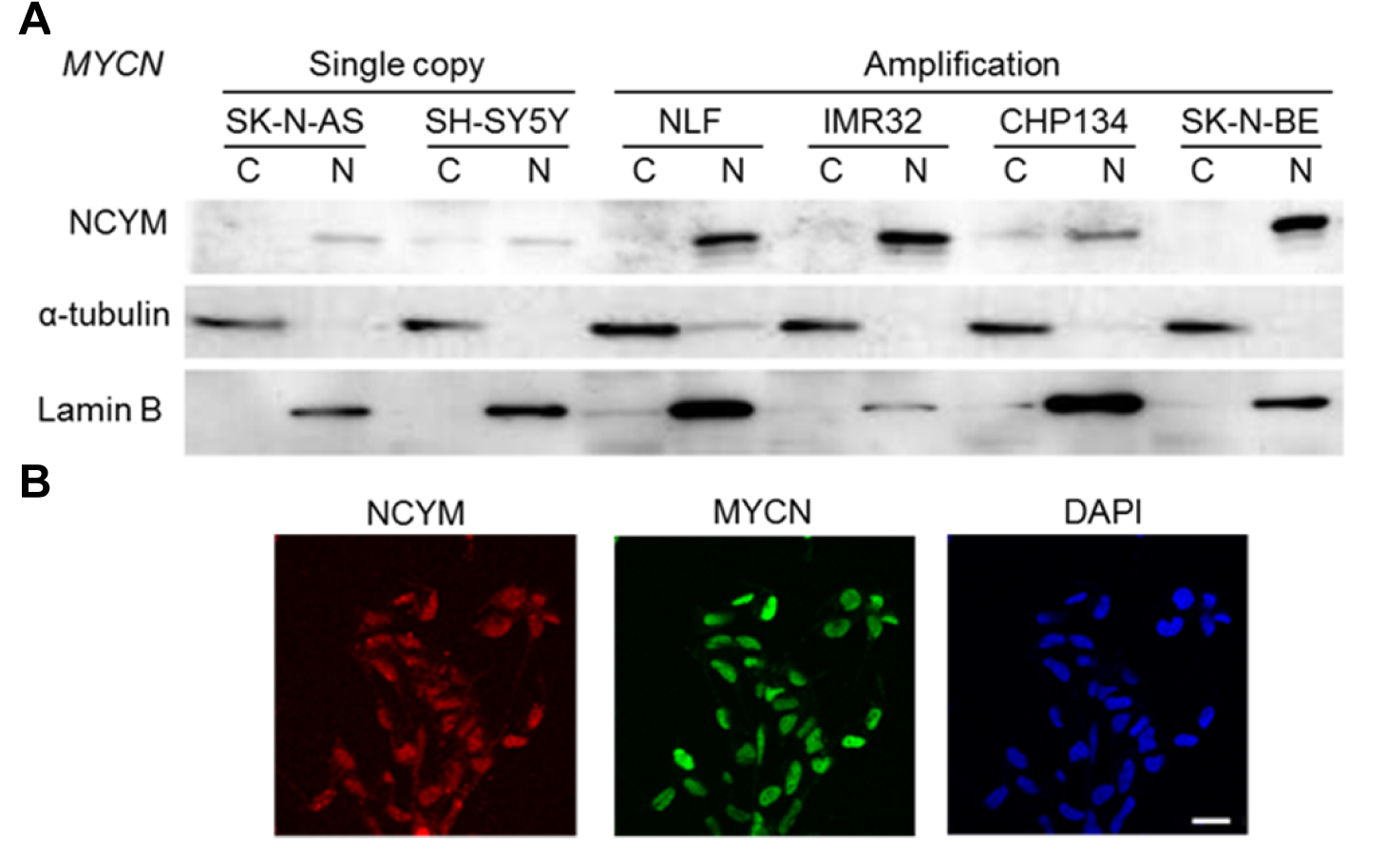

Supplement: Figure S4 — Subcellular localization of NCYM protein in neuroblastoma cells. (A) Localization of NCYM protein in neuroblastoma cells. The indicated neuroblastoma cells were biochemically fractionated into nuclear and cytoplasmic fractions followed by immunoblotting with anti-NCYM antibody. Lamin B and α-tubulin were used as nuclear and cytoplasmic markers, respectively. SK-N-AS and SH-SY5Y are human neuroblastoma cells with a single copy of MYCN, and NLF, IMR32, CHP134, and SK-N-BE are human neuroblastoma cells with amplified MYCN. (B) Nuclear staining of NCYM and MYCN protein in MYCN-amplified human neuroblastoma TGW cells analyzed by confocal fluorescence microscopy. Scale bar, 50 µm. (TIF) [file pgen.1003996.s004.tif]

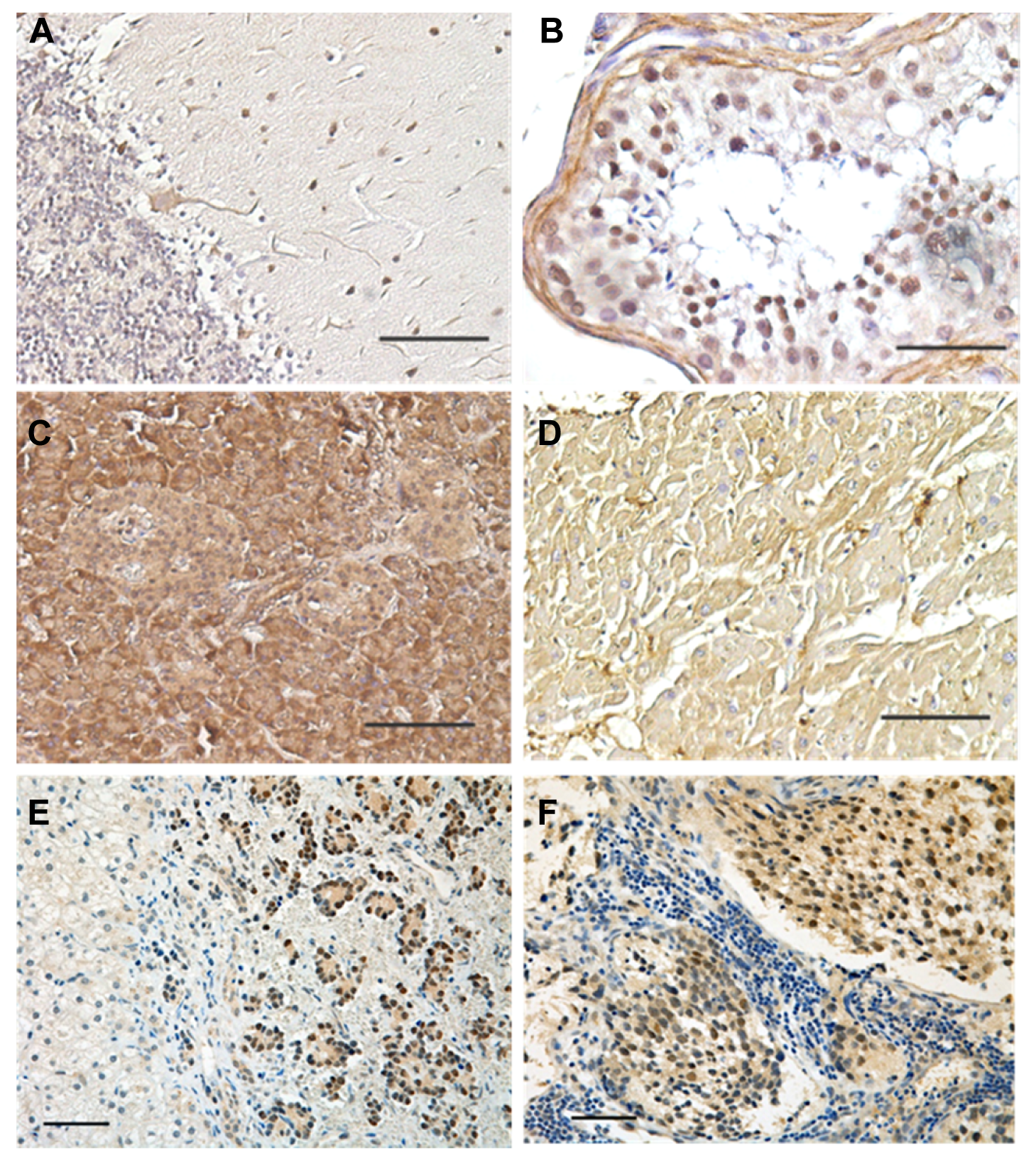

Supplement: Figure S5 — NCYM protein expression in human normal and neuroblastoma tissues analyzed by immunohistochemistry. The indicated human normal tissues (tissue array, FDA808a-1) were stained with anti-NCYM antibody. (A) Cerebellum; scale bar, 100 µm. (B) Testis; scale bar, 50 µm. (C) Pancreas; scale bar, 100 µm. (D) Heart; scale bar, 100 µm. (E) Human metastatic neuroblastoma in the liver (Stage 4S); scale bar, 50 µm. (F) Human metastatic neuroblastoma in the lymph node (Stage 4); scale bar, 50 µm. (TIF) [file pgen.1003996.s005.tif]

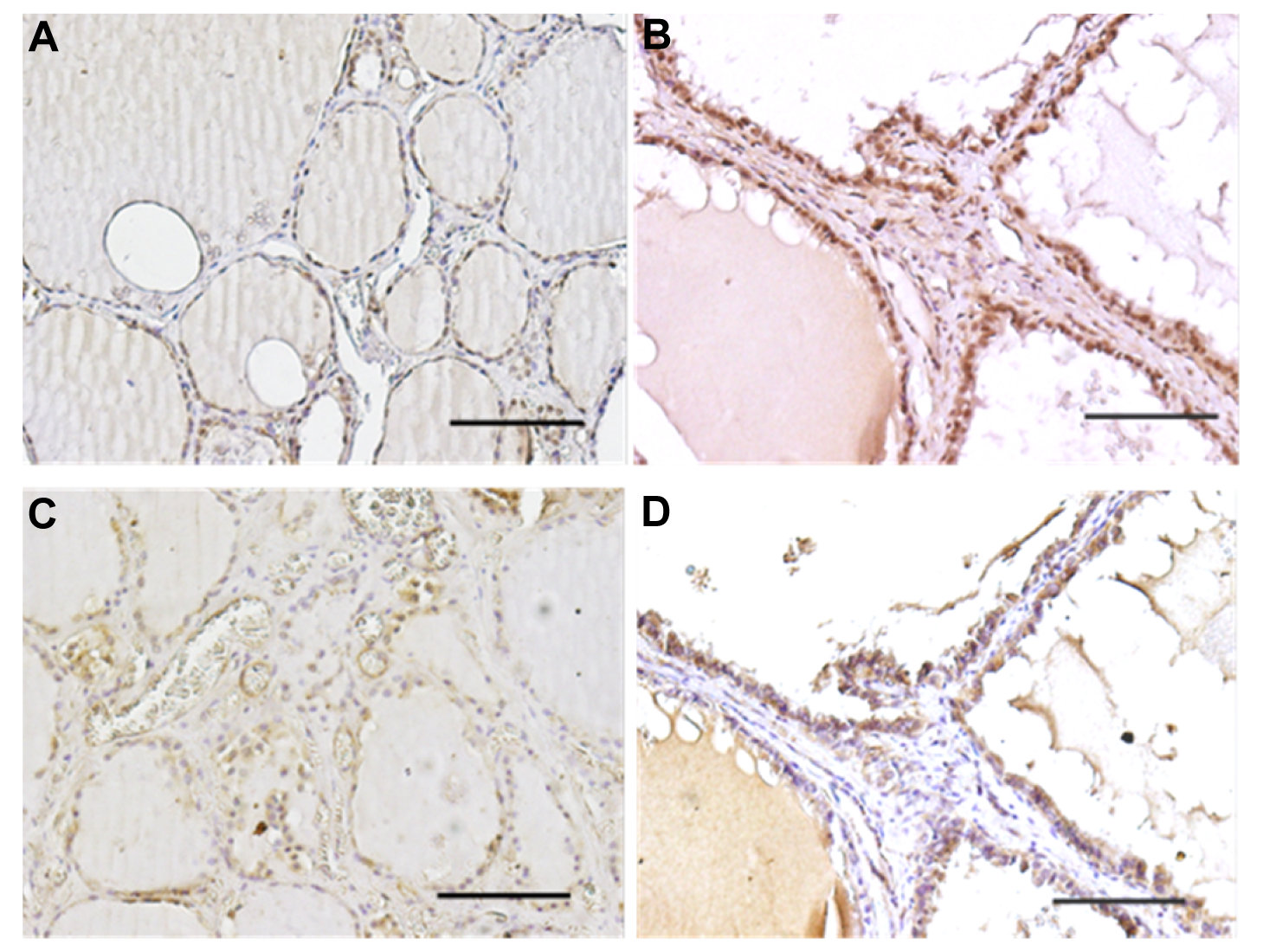

Supplement: Figure S6 — Expression of NCYM and MYCN protein in human thyroid tumors analyzed by immunohistochemistry. Normal and cancerous human tissues (tissue array, FDA808a-2) were stained with anti-NCYM (A and B) or anti-MYCN antibody (C and D). (A), (C), Normal thyroid. (B), (D), Thyroid tumors. Scale bars, 100 µm. (TIF) [file pgen.1003996.s006.tif]

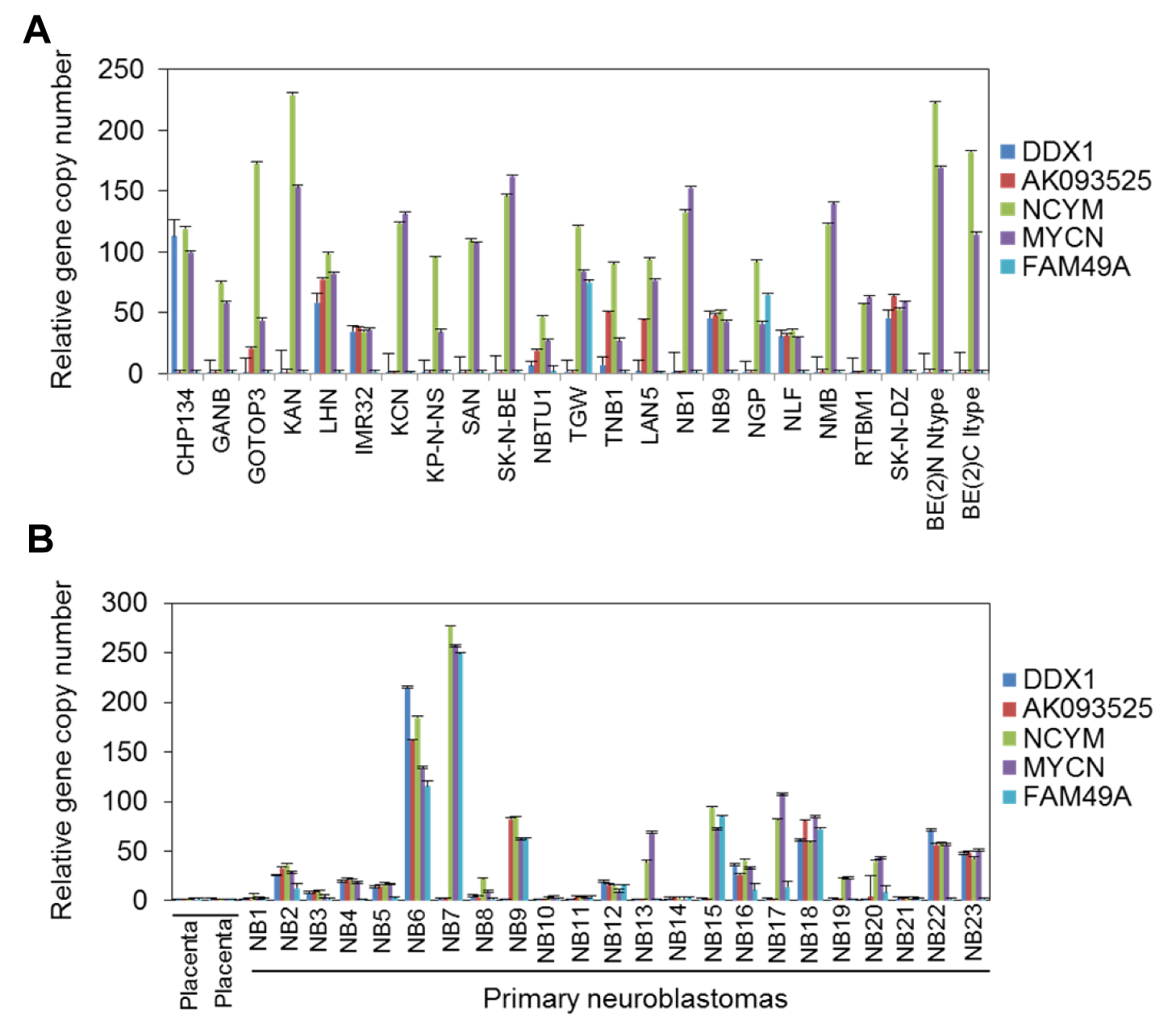

Supplement: Figure S7 — Co-amplification of MYCN and NCYM genes in human neuroblastoma cell lines and primary neuroblastomas. Average gene copy number was calculated based on the signals of multiple probes targeted to the indicated gene in array CGH. Twenty-three MYCN-amplified human neuroblastoma cell lines (A) or 23 human primary neuroblastomas (B) were analyzed by array CGH. (TIF) [file pgen.1003996.s007.tif]

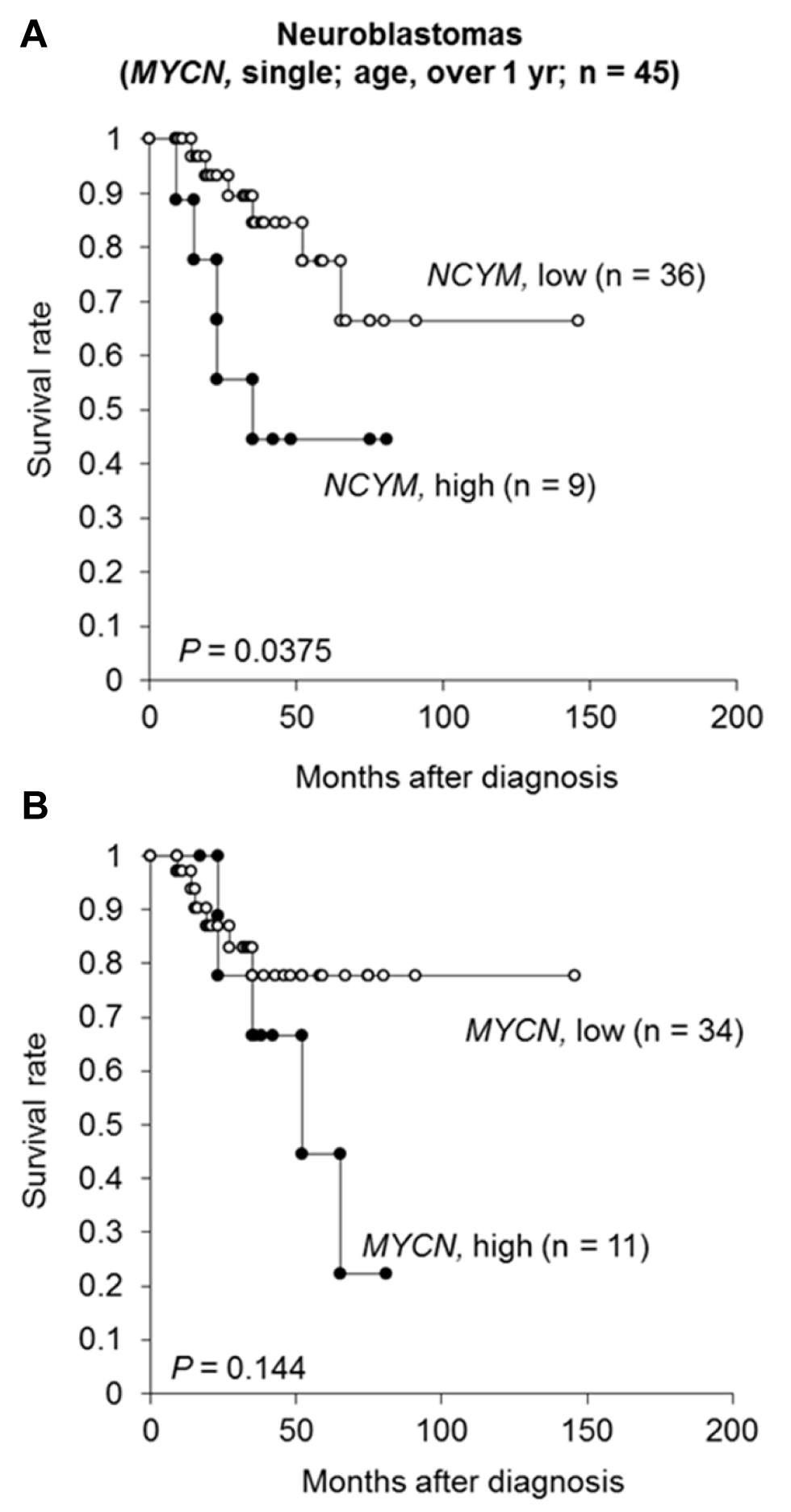

Supplement: Figure S8 — High NCYM mRNA expression is associated with poor prognosis in neuroblastomas without MYCN amplification. MYCN non-amplified neuroblastomas diagnosed at over one year of age were analyzed using Kaplan–Meier survival curves based on the expression levels of NCYM mRNA (A) or MYCN mRNA (B). The expression levels of NCYM or MYCN mRNA were examined by qRT-PCR and normalized by GAPDH. The average of the expression levels was used as a threshold to divide the tumors with low expression (open circle; A, n = 36, B, n = 34) from those with high expression (closed circle; A, n = 9, B, n = 11). P values of (A) and (B) were 0.0375 and 0.144, respectively (Log-rank test). (TIF) [file pgen.1003996.s008.tif]

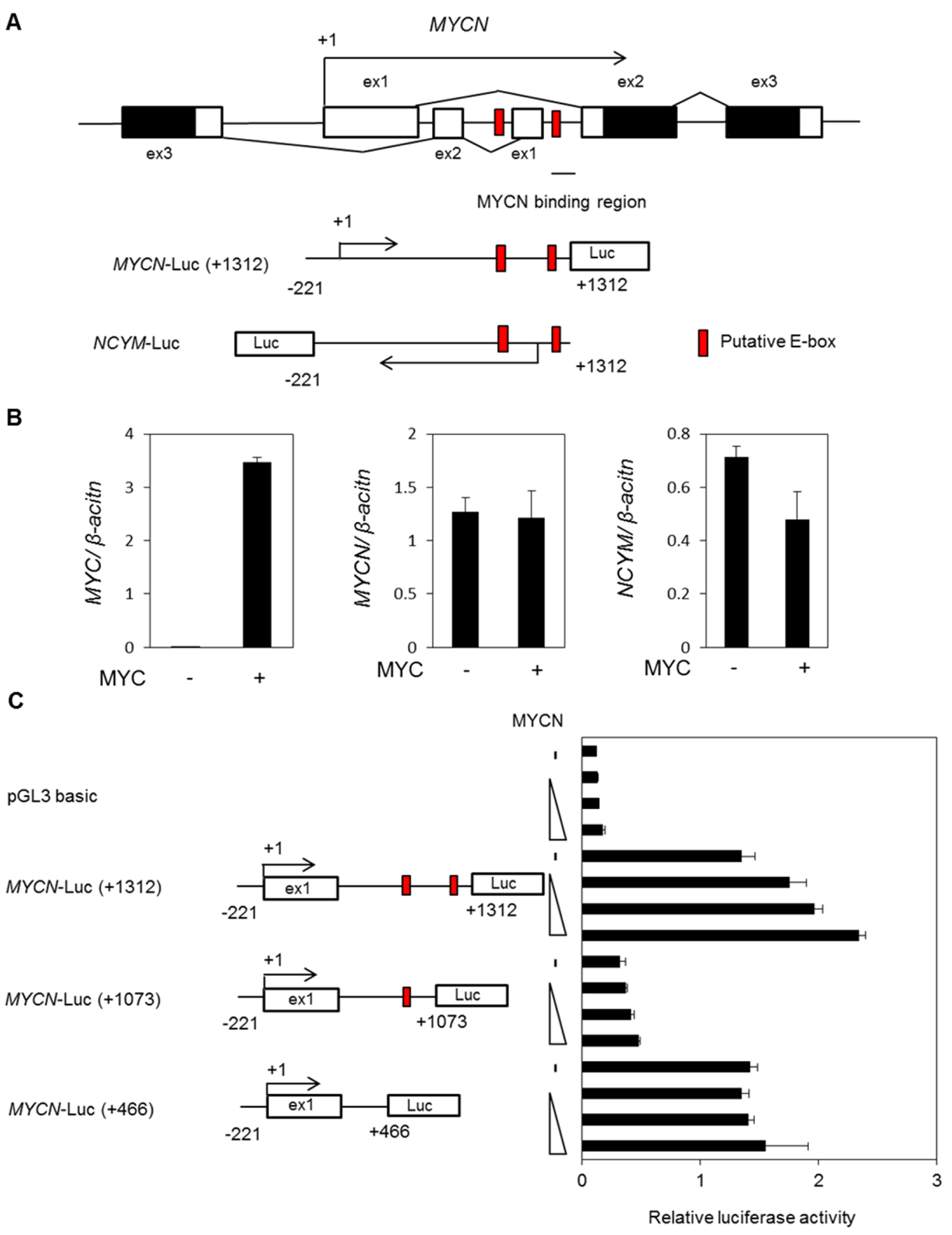

Supplement: Figure S9 — MYCN, but not MYC, activates MYCN transcription in human neuroblastoma cells. (A) Schematic drawing of the MYCN/NCYM promoter region. (B) Relative mRNA levels of MYC, MYCN and NCYM in SK-N-AS MYCN single copy human neuroblastoma cells transfected with 2 µg of a MYC expression vector. mRNA levels were measured by qRT-PCR with β-actin as an internal control. (C) Luciferase reporter assays. SK-N-AS cells were transiently co-transfected with a constant amount of the indicated luciferase reporter constructs bearing various lengths of the human MYCN promoter region (100 ng), a Renilla luciferase reporter plasmid (pRL-TK) (10 ng), and either an empty plasmid (pcDNA3) or with an increasing amount (200, 300, 400 ng) of the expression plasmid for MYCN. Forty-eight hours after transfection, cells were lysed and their luciferase activities were measured. (TIF) [file pgen.1003996.s009.tif]

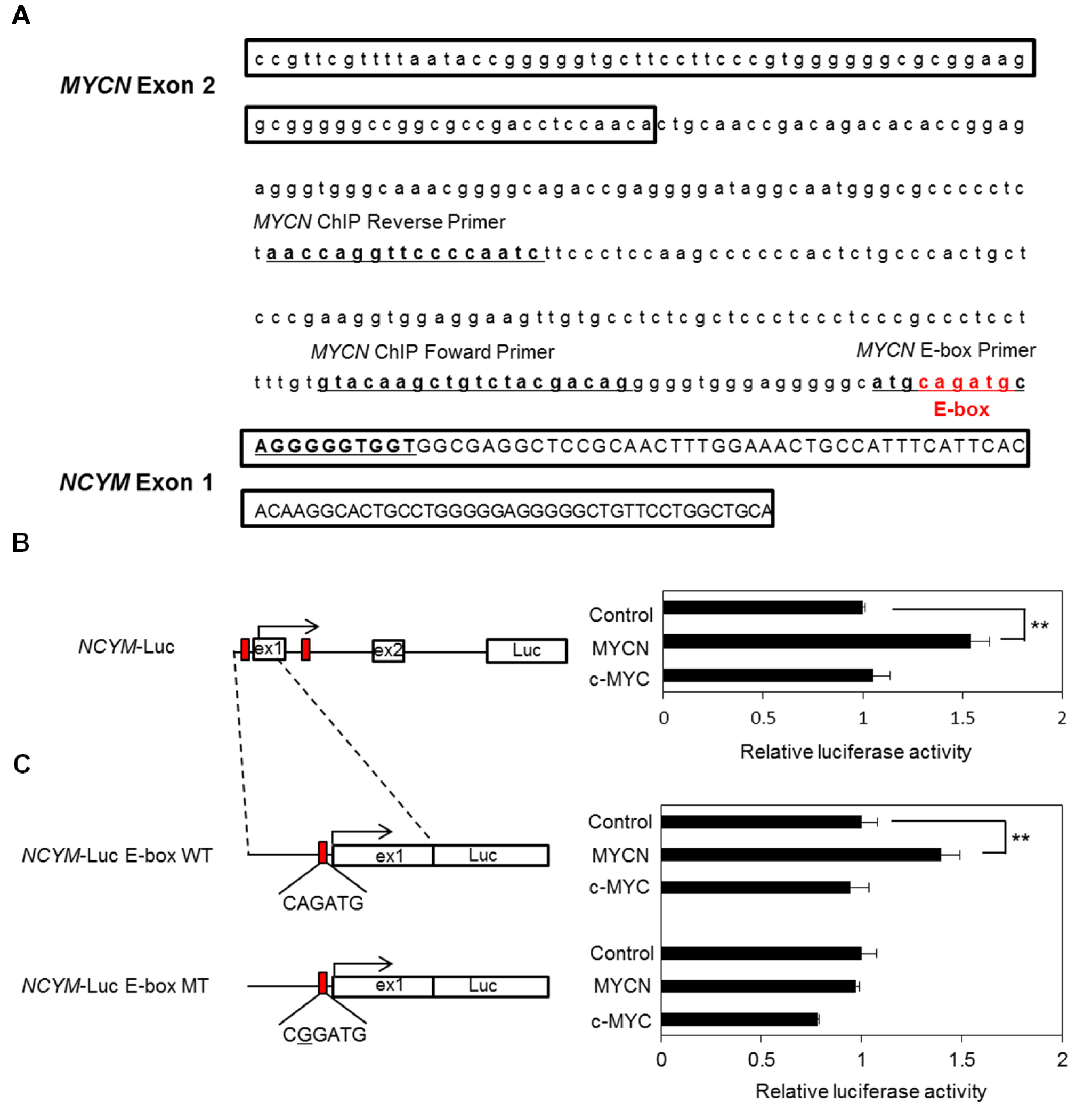

Supplement: Figure S10 — An intact upstream E-box is required for the activation of the NCYM promoter by MYCN. (A) The NCYM promoter sequence. The sequences of primer sets used in our previous report [27] are shown as MYCN ChIP Forward (Reverse) Primer. The recruitment of the MYCN protein to its own intron 1 was detected using those primers. The putative E-box sequence is indicated in red characters. (B) MYCN, but not MYC, enhances NCYM promoter activity. Human neuroblastoma SK-N-AS cells were transfected with 400 ng of the MYCN expression plasmid for 48 hours and then their luciferase activity was measured. (C) The effect of an E-box mutation on MYCN-induced NCYM promoter activity. The WT and mutant NCYM promoters were evaluated for transcriptional activity 24 hours after the transfection of the expression plasmids for MYCN or MYC. The asterisks indicate statistical significance (P<0.01, Student's t-test). (TIF) [file pgen.1003996.s010.tif]

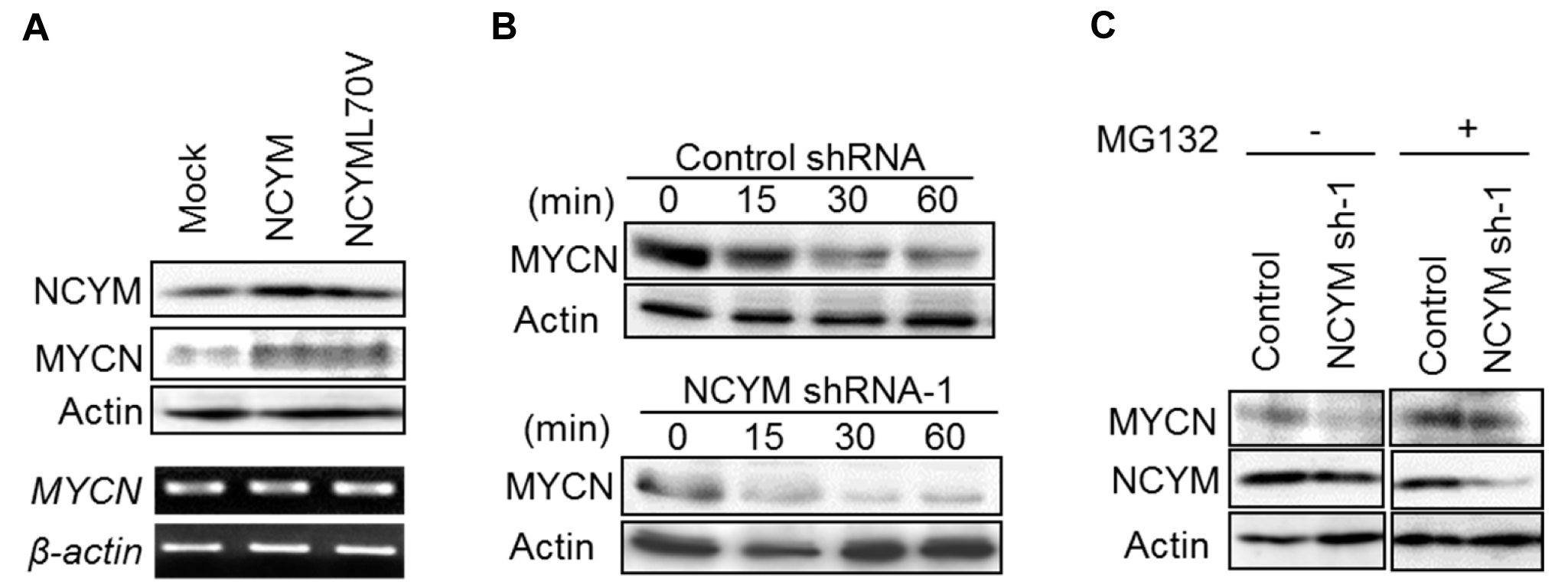

Supplement: Figure S11 — NCYM stabilizes the MYCN protein in the ubiquitin–proteasome system dependent manner. (A) Western blot analysis and RT-PCR. Both NCYM and its SNP type (NCYML70V) induce MYCN expression levels in CHP134 cells. (B) Western blot analysis of MYCN expression in CHP134 cells transfected with NCYM or control shRNA, followed by treatment with 50 µM cycloheximide (CHX), and harvested at the indicated time points. (C) Proteasome inhibitor MG132 treatment of NCYM knockdown CHP134 cells. Western blot analysis showed that NCYM-mediated downregulation of MYCN is inhibited by MG132 treatment. Actin was used as a loading control. (TIF) [file pgen.1003996.s011.tif]

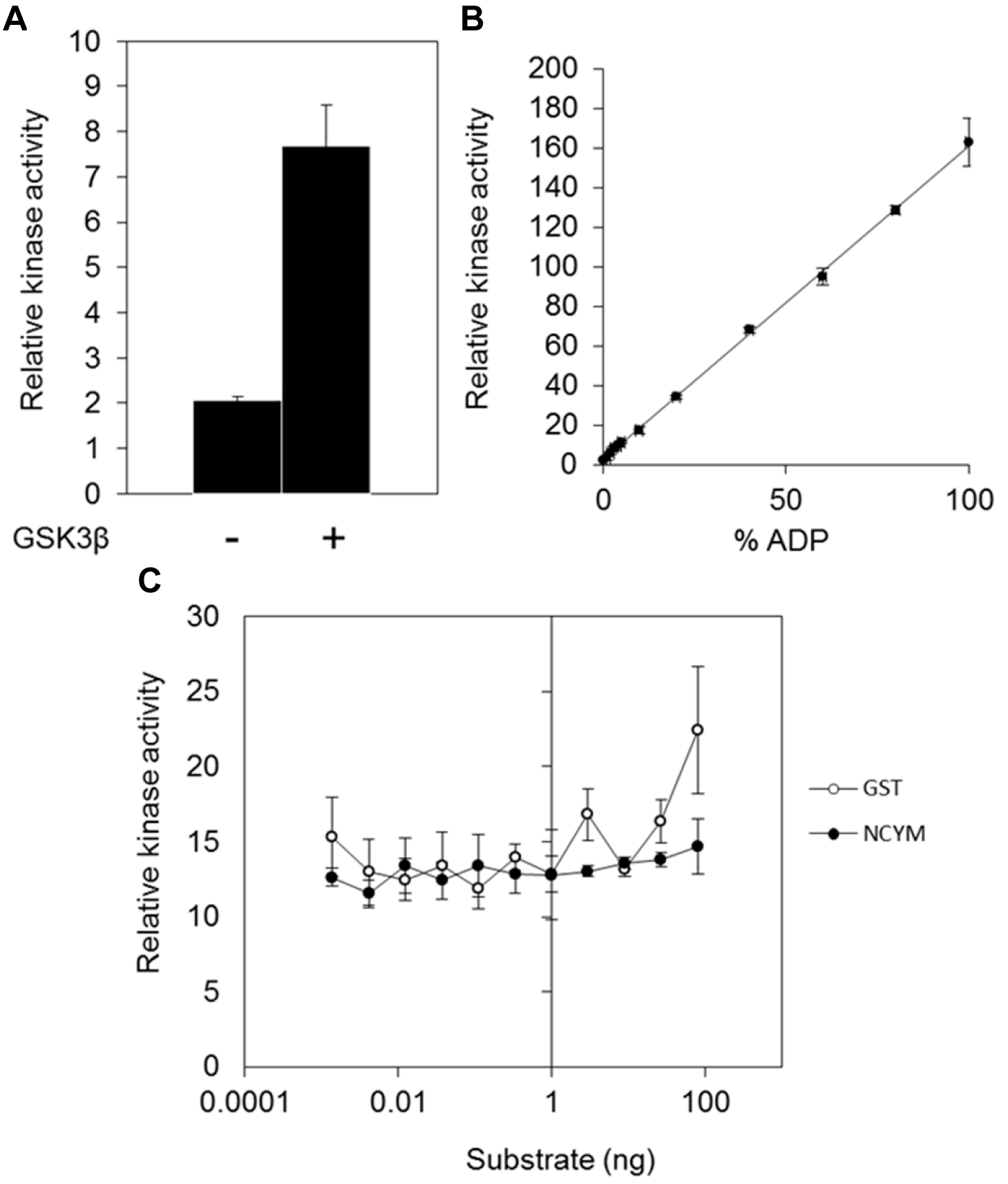

Supplement: Figure S12 — GSK3β does not phosphorylate NCYM protein. (A) In vitro kinase assay. The phosphorylation of the control substrate human glucose synthase 1 by GSK3β was measured to test the assay system. (B) The correlation between the percentage of ADP and the relative kinase activity measured by luciferase activity (R2 = 0.9994). (C) The relative kinase activity of GSK3β was not increased when GST or NCYM were used as a substrate. (TIF) [file pgen.1003996.s012.tif]

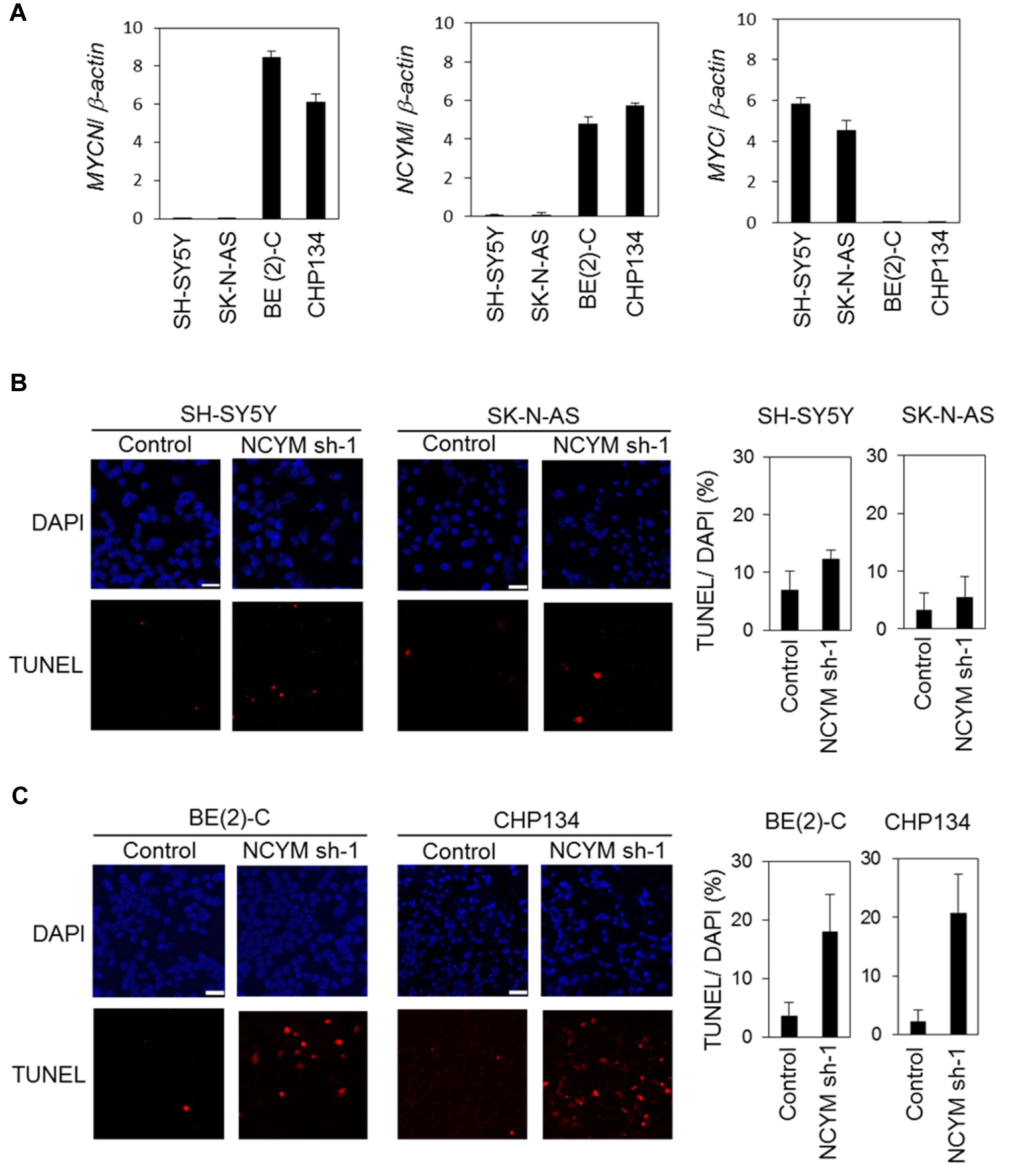

Supplement: Figure S13 — NCYM knockdown promotes apoptosis in MYCN-amplified neuroblastoma cells. (A) The relative mRNA levels of MYCN, NCYM and MYC in human neuroblastoma cells. Levels of mRNA were measured by qRT-PCR with β-actin as an internal control. (B and C) TUNEL staining. The indicated human neuroblastoma cells were lentivirally transfected with the indicated shRNA. Sevently-two hours after the transfection, cells were fixed in 4% paraformaldehyde and subjected to TUNEL staining. Cell nuclei were stained with DAPI (upper panels). Scale bars, 50 µm. The percentage of TUNEL-positive cells was calculated as the average of three different microscopic fields. SH-SY5Y and SK-N-AS are MYCN single copy cells (B), and BE (2)-C and CHP134 are MYCN-amplified cells (C). (TIF) [file pgen.1003996.s013.tif]

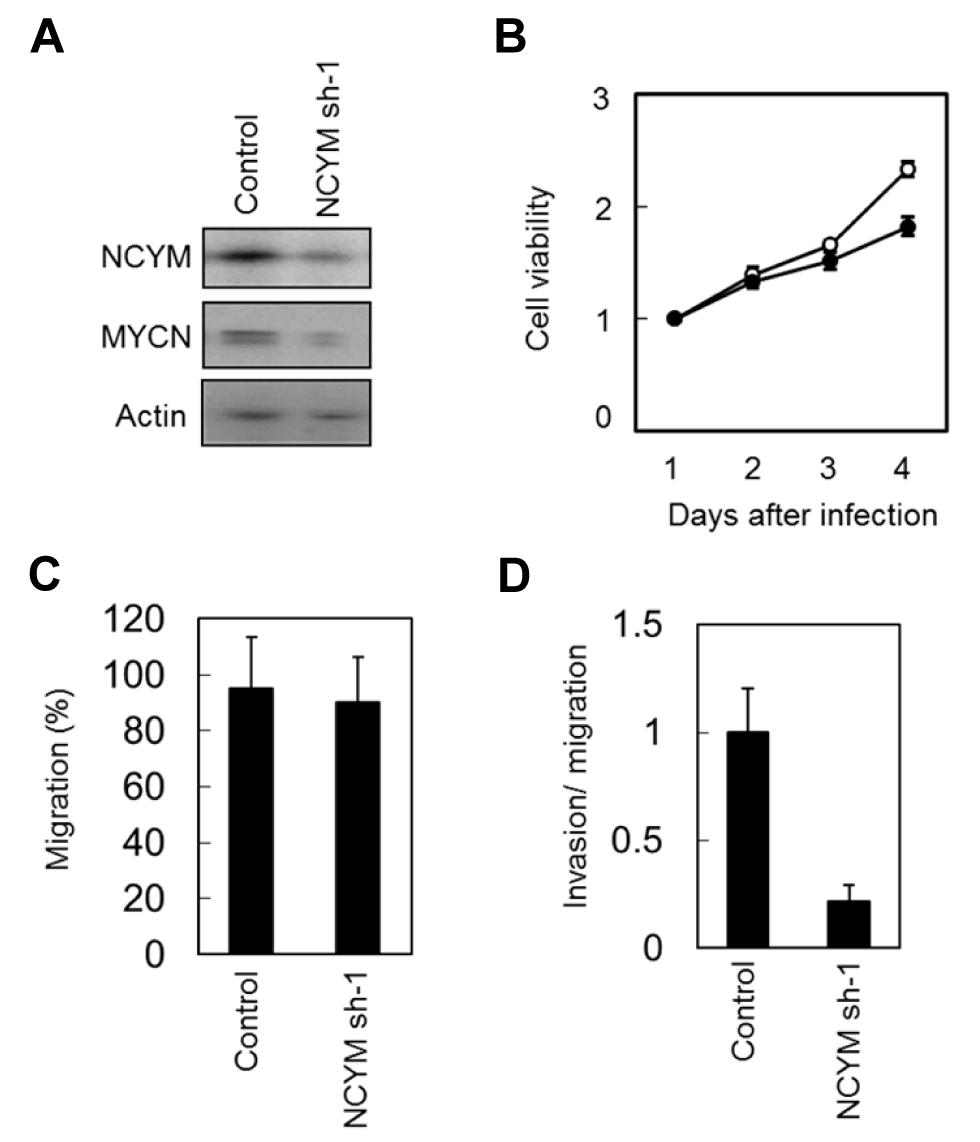

Supplement: Figure S14 — NCYM knockdown inhibits the proliferation and invasion in BE (2)-C cells. (A) Western blot analysis showed that NCYM knockdown decreased MYCN protein in BE (2)-C cells. (B) Cell proliferation assay. After transfection with NCYM shRNA (closed circle) or control shRNA (open circle), cell proliferation was examined in an MTT assay, at the indicated time points. (C, D) the effect of NCYM knockdown on cellular migration (C) and invasion (D). Three days after the introduction of NCYM shRNA, the cells were adjusted to 1×10^5^ cells/ml and subjected to a Boyden chamber invasion assay. (TIF) [file pgen.1003996.s014.tif]

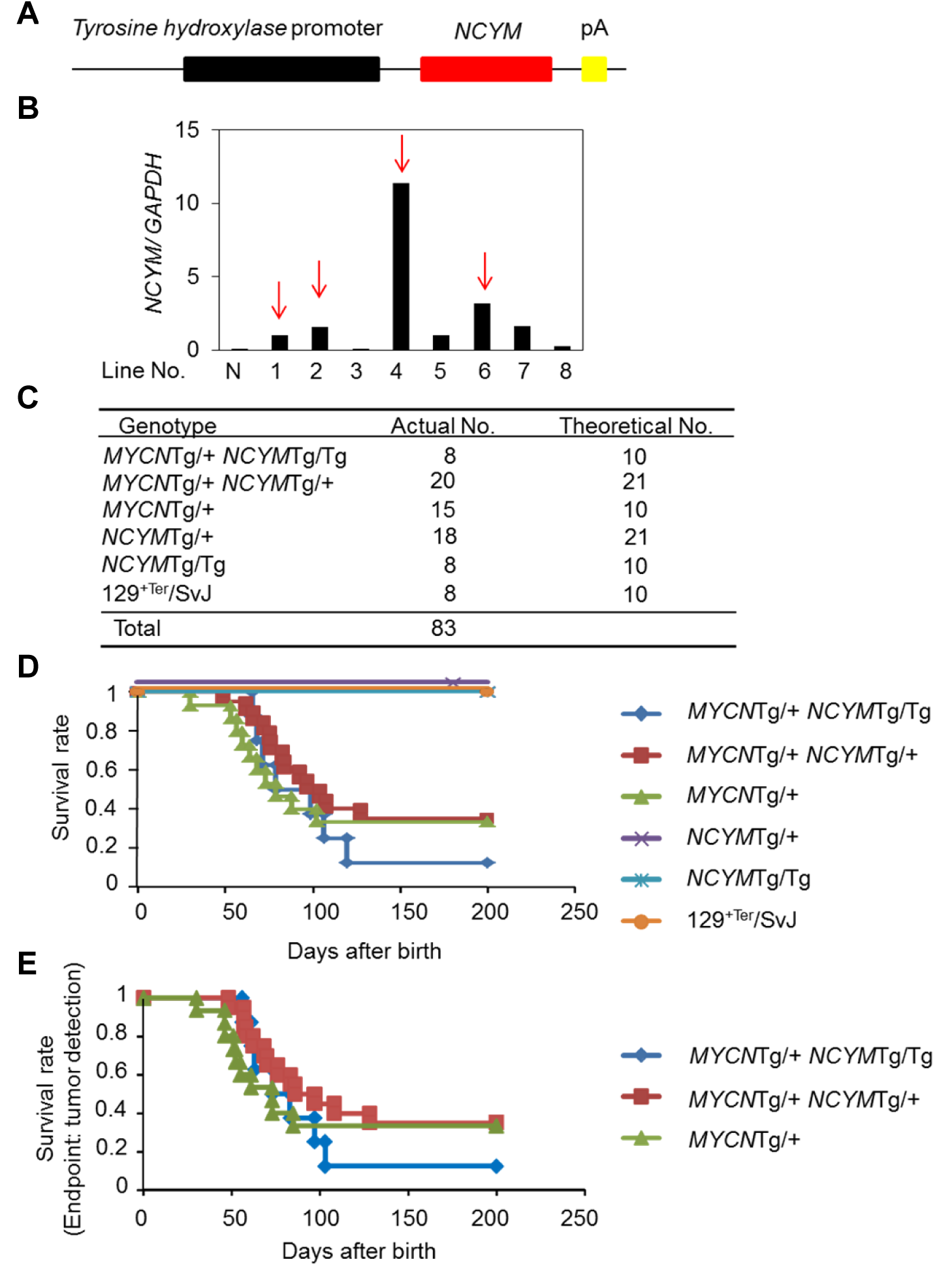

Supplement: Figure S15 — Generation of MYCN/NCYM transgenic mice. (A) A NCYM cDNA was ligated 3′ to the rat TH promoter to generate the pGEM7z(f+)-FLAG-NCYM transgenic constructs. (B) NCYM mRNA expression in the adrenal tissues of NCYM transgenic mice was measured by qRT-PCR. The expression levels were normalized to mouse GAPDH. Red arrows indicate the mouse lines used for further experiments. (C) Table showing the distribution of actual numbers of transgenic mice (line 6) resulting from intercrossing of MYCN Tg/+ NCYM Tg/+ and NCYM Tg/+ and the corresponding theoretical numbers (P>0.05, Chi-square independence test). This result indicates that the NCYM and MYCN transgenes have a marginal effect on the embryonic lethality of mice. (D) Kaplan–Meier survival curves of 83 mice resulting from intercrosses of MYCN Tg/+ NCYM Tg/+ and NCYM Tg/+ (mouse line 6). (E) Kaplan–Meier analysis for tumor incidences in MYCN Tg/+ and MYCN/NCYM Tg mice (mouse line 6). (TIF) [file pgen.1003996.s015.tif]

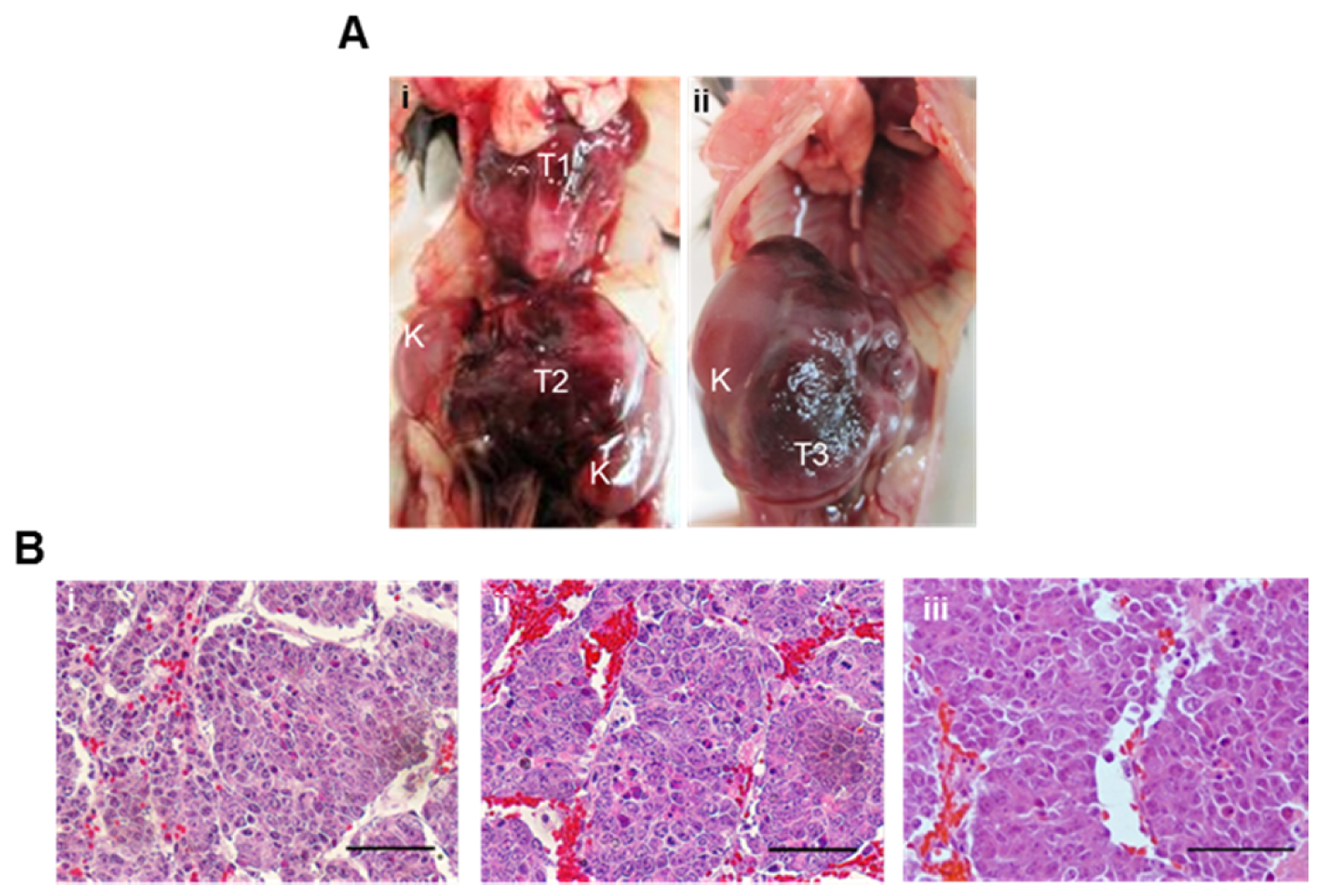

Supplement: Figure S16 — Neuroblastoma histology of MYCN transgenic mice and MYCN/NCYM double transgenic mice. (A) Neuroblastomas arise as primary lesions in a MYCN/NCYM double transgenic mouse (i) and MYCN transgenic mice (ii). Thoracic paraspinal (T1) and abdominal (T2, T3) tumors. K, kidney. (B) H&E staining of T1 (i), T2 (ii), and T3 (iii). (TIF) [file pgen.1003996.s016.tif]

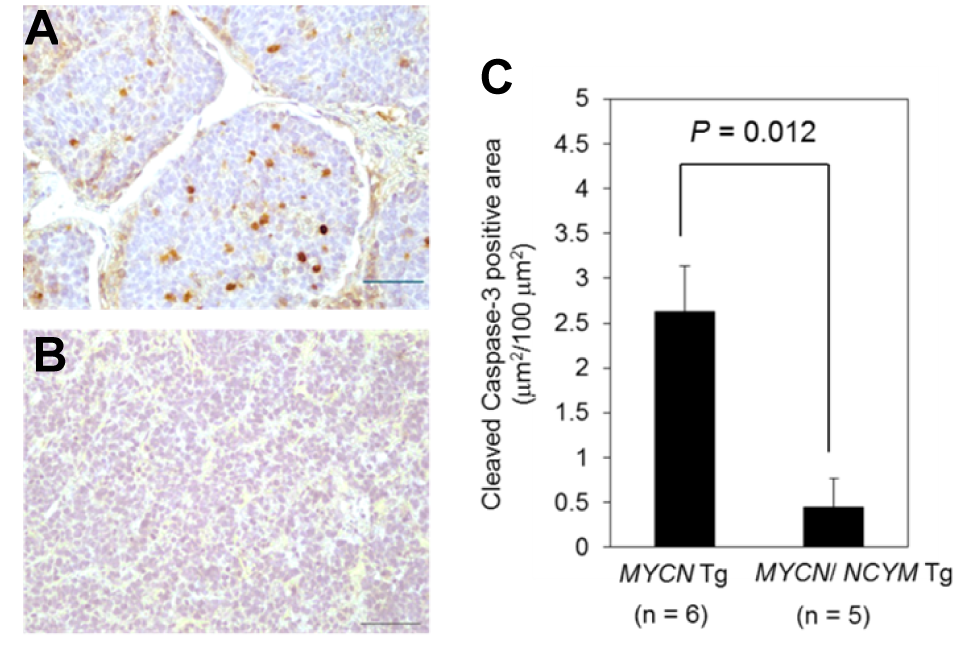

Supplement: Figure S17 — NCYM inhibits apoptosis in the neuroblastomas of MYCN/NCYM double transgenic mice. The number of apoptotic cells in neuroblastomas from MYCN transgenic mice (A) and MYCN/NCYM double transgenic mice (B) were measured using cleaved capase-3 staining. Scale bar, 50 µm. (C) Quantification of cleaved caspase-3–positive areas in the tumors. The apoptotic cells were calculated by averaging the number of cleaved caspase-3–positive areas counted in 5 randomly selected fields (100 µm2) per slide using WinROOF software (version 7.0, Mitani Corp.). P value was 0.012 (Student's t-test). (TIF) [file pgen.1003996.s017.tif]

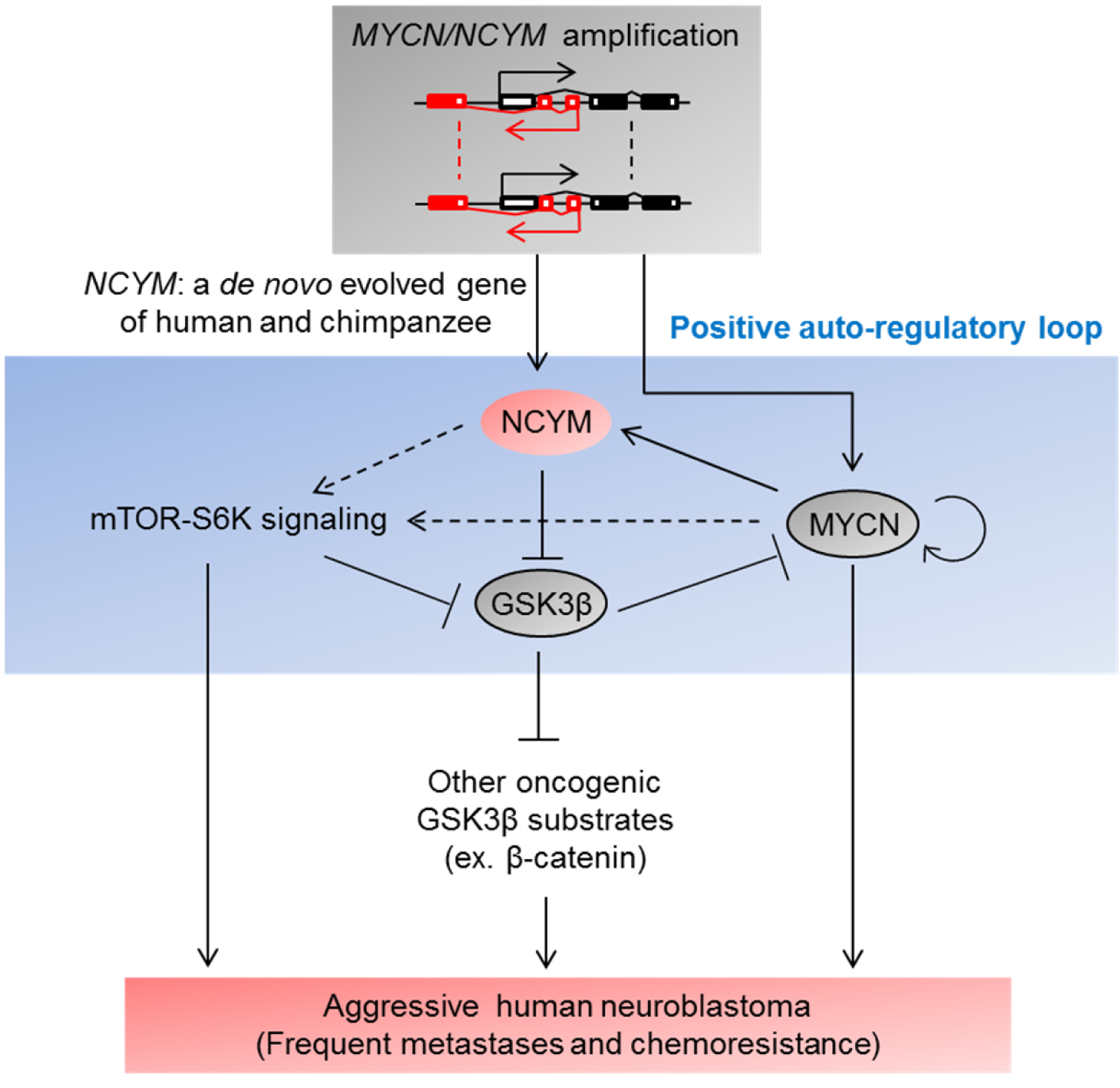

Supplement: Figure S18 — Schematic model of NCYM function in aggressive human neuroblastomas. (TIF) [file pgen.1003996.s018.tif]
