# Supplementary material for: NCYM, a Cis-Antisense Gene of MYCN, Encodes a De Novo Evolved Protein That Inhibits GSK3β Resulting in the Stabilization of MYCN in Human Neuroblastomas
Source: PLoS Genet. 2014 Jan 2;10(1):e1003996. doi: 10.1371/journal.pgen.1003996 (PMC3879166; doi:10.1371/journal.pgen.1003996)
Supplement: Table S1 — Correlation between the expression of NCYM or MYCN and other prognostic factors. (DOC) [file pgen.1003996.s019.doc]

**Table S1 Correlation between the expression of *NCYM* or *MYCN* and other prognostic factors.**

|  |  | ***NCYM*** | | ***MYCN*** | |
| --- | --- | --- | --- | --- | --- |
| **Variable** | **No.** | **MeanSEM** | ***P* value** | **Mean$\pm$SEM** | ***P* value** |
| **Age, y** |  |  |  |  |  |
| <1 | 43 | 80.31 $\pm$ 20.79 | 0.078 | 2.46 0.97 | 0.097 |
| ≥1 | 63 | 385.43 140.76 |  | 8.42 2.86 |  |
| ***MYCN* copy number** |  |  |  |  |  |
| Single copy | 86 | 32.10 4.08 | <0.001 | 0.75 0.09 | <0.001 |
| Amplified | 20 | 1248.75 385.30 |  | 28.60 7.59 |  |
| **Tumor stage** |  |  |  |  |  |
| 1, 2, 4s | 49 | 72.62 25.62 | 0.039 | 1.34 0.35 | 0.014 |
| 3, 4 | 57 | 424.15 154.05 |  | 10.02 3.18 |  |
| **DNA index** |  |  |  |  |  |
| Aneuploid | 43 | 76.27 39.45 | 0.068 | 2.37 1.40 | 0.069 |
| Diploid | 48 | 354.68 138.01 |  | 9.43 3.42 |  |
| **Shimada pathology** |  |  |  |  |  |
| Favorable | 62 | 84.72 26.06 | 0.003 | 2.41 0.83 | 0.004 |
| Unfavorable | 30 | 698.88 282.50 |  | 14.50 5.61 |  |
| ***TrkA* expression** |  |  |  |  |  |
| Low | 50 | 500.06 174.75 | 0.009 | 11.01 3.59 | 0.008 |
| High | 53 | 46.81 10.79 |  | 1.49 0.47 |  |
| **Tumor origin** |  |  |  |  |  |
| Adrenal gland | 55 | 452.03 159.73 | 0.020 | 10.05 3.23 | 0.016 |
| Others | 51 | 57.42 16.75 |  | 1.65 0.81 |  |
