# Supplementary material for: NCYM, a Cis-Antisense Gene of MYCN, Encodes a De Novo Evolved Protein That Inhibits GSK3β Resulting in the Stabilization of MYCN in Human Neuroblastomas
Source: PLoS Genet. 2014 Jan 2;10(1):e1003996. doi: 10.1371/journal.pgen.1003996 (PMC3879166; doi:10.1371/journal.pgen.1003996)
Supplement: Table S2 — Multiple Cox regression analyses of NCYM expression, MYCN expression, age, MYCN amplification, stage, DNA index, Shimada pathology, TrkA expression, and origin. (DOC) [file pgen.1003996.s020.doc]

**Table S2 Multiple Cox regression analyses of *NCYM* expression, *MYCN* expression, age, *MYCN* amplification, stage, DNA index, Shimada pathology, *TrkA* expression, and origin.**

| **Factor** | **No.** | ***P* value** | **HR (95% CI)** |
| --- | --- | --- | --- |
| **Univariate analysis** |  |  |  |
| *NCYM* mRNA expression (high vs. low) | 106 | 0.0014 | 4.70 (1.91–10.53) |
| *MYCN* mRNA expression (high vs. low) | 106 | 0.0006 | 4.98 (2.10–11.01) |
| Age (≥1 vs. <1y) | 106 | 0.0015 | 4.39 (1.68–14.97) |
| *MYCN* amplification (amplified vs. single copy) | 106 | <0.0001 | 6.10 (2.81–13.18) |
| Stage (1, 2, 4s vs. 3, 4) | 106 | <0.0001 | 6.12 (2.35–20.94) |
| DNA index (diploid vs. aneuploid) | 91 | <0.0001 | 10.98 (3.13–69.55) |
| Shimada pathology (unfavorable vs. favorable) | 92 | 0.0149 | 3.19 (1.25–8.67) |
| *TrkA* expression (high vs. low) | 103 | 0.0004 | 4.43 (1.89–12.12) |
| Origin (adrenal gland vs. others) | 106 | 0.0142 | 2.68 (1.21–6.50) |
| **Multivariate analysis** |  |  |  |
| *NCYM* mRNA expression (high vs. low) | 106 | 0.9372 | 1.09 (0.06–6.01) |
| *MYCN* mRNA expression (high vs. low) | 106 | 0.1940 | 5.37 (0.30–26.78) |
|  |  |  |  |
| *NCYM* mRNA expression (high vs. low) | 106 | 0.0095 | 3.44 (1.39–7.88) |
| Age (≥12 vs. <12 m) | 106 | 0.0101 | 3.55 (1.32–12.31) |
|  |  |  |  |
| *NCYM* mRNA expression (high vs. low) | 106 | 0.0056 | 3.82 (1.52–8.80) |
| Stage (1, 2, 4s vs. 3, 4) | 106 | 0.0003 | 5.52 (2.08–19.02) |
|  |  |  |  |
| *NCYM* mRNA expression (high vs. low) | 106 | 0.9810 | 1.01 (0.30–3.06) |
| *MYCN* amplification (amplified vs. single copy) | 106 | 0.0032 | 6.15 (1.08–16.15) |
|  |  |  |  |
| *NCYM* mRNA expression (high vs. low) | 106 | 0.1420 | 2.17 (0.75–5.57) |
| DNA index (diploid vs. aneuploid) | 91 | 0.0003 | 9.20 (2.52–59.14) |
|  |  |  |  |
| *NCYM* mRNA expression (high vs. low) | 106 | 0.0325 | 2.77 (1.10–6.51) |
| *TrkA* expression (high or low) | 103 | 0.0065 | 3.48 (1.40–9.84) |
|  |  |  |  |
| *NCYM* mRNA expression (high vs. low) | 106 | 0.0131 | 3.43 (1.32–8.53) |
| Origin (adrenal tissues vs. others) | 106 | 0.1601 | 1.90 (0.78–4.90) |
