# Supplementary material for: NCYM, a Cis-Antisense Gene of MYCN, Encodes a De Novo Evolved Protein That Inhibits GSK3β Resulting in the Stabilization of MYCN in Human Neuroblastomas
Source: PLoS Genet. 2014 Jan 2;10(1):e1003996. doi: 10.1371/journal.pgen.1003996 (PMC3879166; doi:10.1371/journal.pgen.1003996)
Supplement: Table S3 — Summary of mice bearing neuroblastomas. (DOC) [file pgen.1003996.s021.doc]

**Table S3** Summary of mice bearing neuroblastomas

*

*M54 developed a single tumor in the stomach, but the primary tumor was not detectable. Therefore, the tumor was not counted as a macroscopic metastatic tumor in Figure 4C.
